# LINC00606 promotes glioblastoma progression through sponge miR-486-3p and interaction with ATP11B

**DOI:** 10.1186/s13046-024-03058-z

**Published:** 2024-05-09

**Authors:** Naijun Dong, Wenxin Qi, Lingling Wu, Jie Li, Xueqi Zhang, Hao Wu, Wen Zhang, Jiawen Jiang, Shibo Zhang, Wenjun Fu, Qian Liu, Guandong Qi, Lukai Wang, Yanyuan Lu, Jingyi Luo, Yanyan Kong, Yihao Liu, Robert Chunhua Zhao, Jiao Wang

**Affiliations:** 1 School of Life Sciences, Shanghai University, Shanghai, 200444 P. R. China; 2 School of Medicine, Shanghai University, Shanghai, China; 3 Shanghai Institute of Phage, Shanghai Public Health Clinical Center, Fudan University, Shanghai, China; 4 Institute of Basic Medical Sciences Chinese Academy of Medical Sciences, School of Basic Medicine Peking, Union Medical College, Beijing, China; 5 Centre of Excellence in Tissue Engineering, Chinese Academy of Medical Sciences, Peking Union Medical College, Beijing, China; 6 Beijing Key Laboratory of New Drug Development and Clinical Trial of Stem Cell Therapy (BZ0381), Beijing, China; 7 Residential College, Shanghai University, Shanghai, China; 8 Department of General Surgery, Ruijin Hospital, Shanghai Jiaotong University School of Medicine, Shanghai, 200025 China; 9 PET Center, Huashan Hospital, Fudan University, Shanghai, China

**Keywords:** Glioblastoma (GBM), LINC00606, miR-486-3p, TCF12, ATP11B

## Abstract

**Background:**

LncRNAs regulate tumorigenesis and development in a variety of cancers. We substantiate for the first time that LINC00606 is considerably expressed in glioblastoma (GBM) patient specimens and is linked with adverse prognosis. This suggests that LINC00606 may have the potential to regulate glioma genesis and progression, and that the biological functions and molecular mechanisms of LINC00606 in GBM remain largely unknown.

**Methods:**

The expression of LINC00606 and ATP11B in glioma and normal brain tissues was evaluated by qPCR, and the biological functions of the LINC00606/miR-486-3p/TCF12/ATP11B axis in GBM were verified through a series of in vitro and in vivo experiments. The molecular mechanism of LINC00606 was elucidated by immunoblotting, FISH, RNA pulldown, CHIP-qPCR, and a dual-luciferase reporter assay.

**Results:**

We demonstrated that LINC00606 promotes glioma cell proliferation, clonal expansion and migration, while reducing apoptosis levels. Mechanistically, on the one hand, LINC00606 can sponge miR-486-3p; the target gene TCF12 of miR-486-3p affects the transcriptional initiation of LINC00606, PTEN and KLLN. On the other hand, it can also regulate the PI3K/AKT signaling pathway to mediate glioma cell proliferation, migration and apoptosis by binding to ATP11B protein.

**Conclusions:**

Overall, the LINC00606/miR-486-3p/TCF12/ATP11B axis is involved in the regulation of GBM progression and plays a role in tumor regulation at transcriptional and post-transcriptional levels primarily through LINC00606 sponging miR-486-3p and targeted binding to ATP11B. Therefore, our research on the regulatory network LINC00606 could be a novel therapeutic strategy for the treatment of GBM.

**Graphical Abstract:**

LINC00606 is highly expressed in GBM patients with carcinogenic function and correlated with poor prognosis. LINC00606 regulates glioblastoma progression by sponging miR-486-3p and interacting with ATP11B.

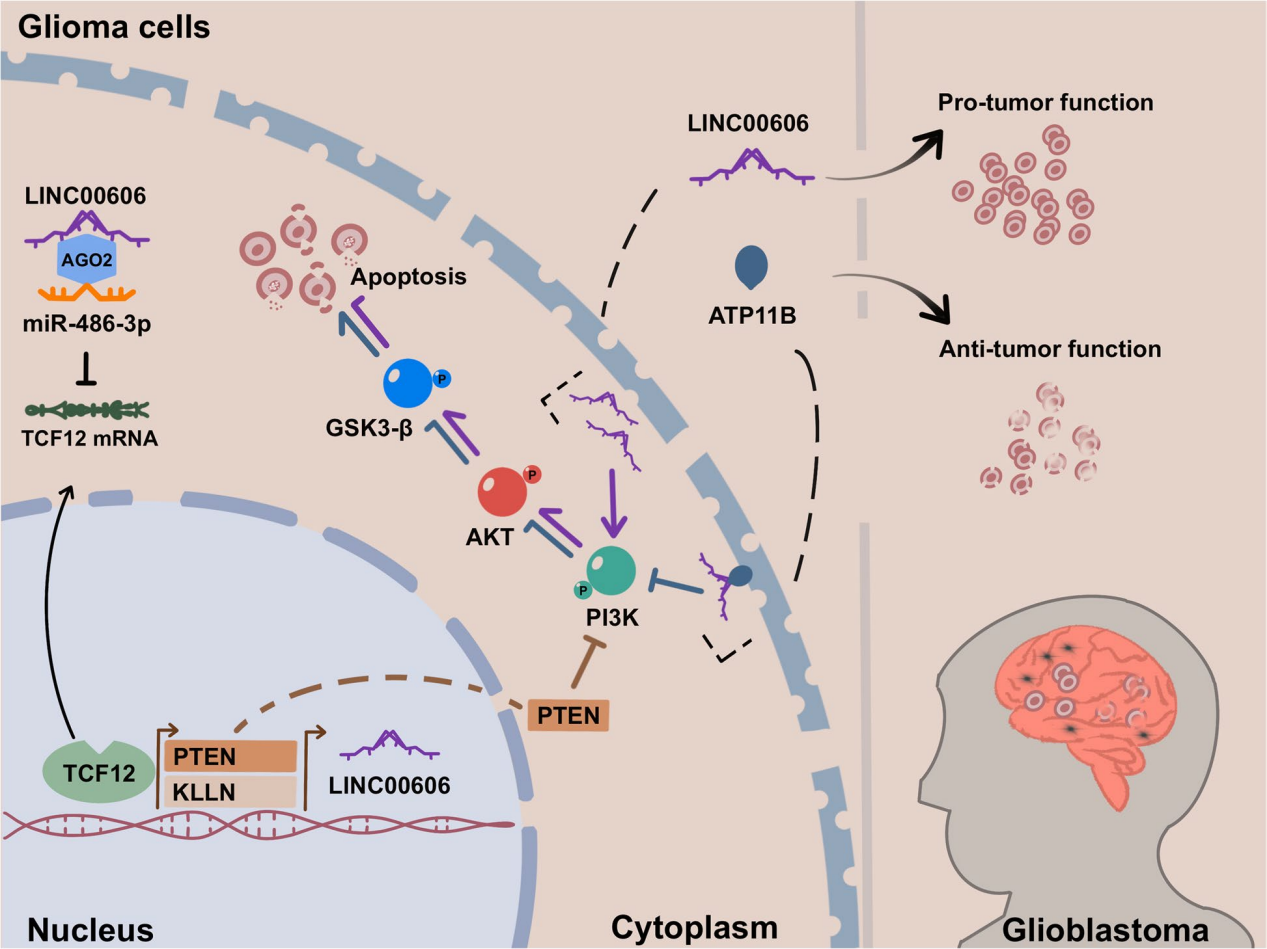

**Supplementary Information:**

The online version contains supplementary material available at 10.1186/s13046-024-03058-z.

## Background

Owing to advancements in medical technology and clinical treatment guidelines, progress has been made in predicting regulatory molecules and optimizing treatment strategies for glioblastoma (GBM); however, prognosis remains poor, and the target genes for effective intervention in GBM progression need to be further explored [1–4]. There is an urgent need to uncover the potential molecular mechanisms involved in glioma progression with a view to improving therapeutics [5, 6]. Mediating apoptosis in cancer intervention and treatment has been recognized clinically, as aberrant activation of apoptosis may exacerbate uncontrolled tumorigenesis and cancer development, in addition to chemotherapy and radiation resistance [7, 8]. Therefore, it is of great significance to explore the key targets regulating the apoptotic process of glioma cells to optimize clinical treatment strategies.

Long non-coding RNAs (LncRNAs), molecules longer than 200 nucleotides devoid of discernible coding potential [9, 10], feature in numerous biological phenomena in tumor cells such as immune response, cancer initiation, and development [11–13]. LncRNAs participate in diverse cellular actions, including epigenetic inheritance, transcription, and post-transcriptional regulation [14, 15]. Additionally, they are employed as molecular sponges to manipulate the expression of target genes by sponging microRNA (miRNA), thereby serving as a regulatory system for tumor suppressor or pro-tumor factors [16, 17]. As crucial players within gene expression networks, lncRNAs interact with specific proteins to regulate cancer cell homeostasis, encompassing proliferation, survival, migration, and other cellular procedures [18–20]. The accompanying mechanism of glioma regulation by lncRNAs remains to be unraveled.

In order to investigate the influence of abnormally expressed lncRNAs in GBM as a gene regulatory center on the occurrence of GBM, we combined clinical samples and sample sequencing datasets to screen target lncRNA. Among them, we refer to the World Health Organization Classification of Tumors of the Central Nervous System, fifth edition (WHO CNS5) [21]. The classification of central nervous system tumors is mainly based on histology, imaging examination, etc., while molecular biomarkers are becoming increasingly important in providing auxiliary and definitive diagnostic information [22, 23], which helps us to carry out pathological screening and further diagnosis of clinical samples. Within the current study, we identify a novel lncRNA, LINC00606, demonstrating that its expression is amplified in GBM and low-grade glioma (LGG), exerting an oncogenic function and being positively correlated with unfavorable prognosis. This corroborates with our observations that downregulation of LINC00606 suppresses tumorigenesis of xenotransplanted glioma cells in vivo. Mechanistically, LINC00606 serves as a sponge for miR-486-3p to diminish its suppressive impact on TCF12. Intriguingly, TCF12, as a transcriptional regulator, is involved in controlling transcriptional initiation of LINC00606, tumor suppressor gene PTEN, and apoptotic gene KLLN. Additional inquiry unveiled that LINC00606 binding to ATP11B interrupts PI3K/AKT signal transduction and reduces glioma cell apoptosis. Thus, elucidation of the regulatory mechanism underlying the LINC00606/miR-486-3p/TCF12/ATP11B axis may lay the foundation for the discovery of new therapeutic strategies for GBM.

## Materials and methods

### Patients and specimens

The patients were retrospectively selected from 120 consecutive patients ≥ 18 years old with newly diagnosed GBMs operated at Zhongshan Hospital, Fudan University, between January 2008 and May 2018. Selection criteria were (1) ≥ 2 preoperative T1-weighted contrast (gadolinium) enhancing (T1wGd) magnetic resonance imaging (MRI) scans taken ≥ 14 days apart and (2) histopathologically verified diagnosis after the 2021 WHO classification. Exclusion criteria were gliomatosis cerebri and non-contrast-enhancing tumors. The IDH mutation status has been checked with immunohistochemistry for IDH-R132H. The diagnosis of glioma was confirmed by Pathologists. Detailed patient information is presented in Supplementary Table S1.

### Cell culture

HEK293T, HEB, U-251MG (U251), U-118MG (U118), U87MG (U87) cells were purchased from the Cell Bank of the Chinese Academy of Sciences (Chinese Academy of Sciences, Shanghai, China). Cells were cultured in DMEM/High Glucose (HyClone, USA) supplemented with 10% FBS and 1% penicillin/streptomycin at 5% CO_2_ at 37 °C. All cell lines were routinely screened for mycoplasma contamination by MycAwayTM Plus-Color One-Step Mycoplasma Detection Kit (Yeasen, Shanghai, China).

### Cell transfection, plasmid construction and lentiviral infection

Cell transfection using Lipo2000™ reagent (Invitrogen, Carlsbad, CA, USA) according to the manufacturer’s instructions. Construction of the ATP11B overexpression plasmid from the empty vector (pCDNA 3.1) is described in our previous work [24, 25]. For the knockdown of ATP11B, cells were transfected with specific small-interfering RNA (siRNAs) or control siRNA synthesized by RiboBio (RiboBio, GuangZhou, China) using Lipo2000™ reagent (Invitrogen, Carlsbad, CA, USA) according to the manufacturer’s instructions. The miR-486-3p inhibitor and miR-486-3p mimic were also synthesized by RiboBio. For the knockdown of LINC00606, specific siRNA and control siRNA were synthesized by Tsingke (Tsingke Biotechnology, Shanghai, China). For overexpression, cDNA encoding human ATP11B (NM 014616) in the pCAG-HAC plasmid was kindly provided by Dr. Hye-Won Shin (Kyoto University). Taking the transfection system of six well plates per well cell as an example, the cell seed plate was performed one day before transfection, so that the density of adherent cells reached 50–60% the next day. During the transfection process, the transfection system was configured and the plasmid concentration per well system was 2 μg. The mimic, inhibitor, or siRNA are 50 nM. After 8 h of transfection, the medium was renewed and the cells were incubated for a further 48 h, after which the cells were harvested and used for subsequent experiments. The lentiviral vectors (OE-ATP11B, Vector, sh-NC and sh-LINC00606) were synthesized by Tsingke (Tsingke Biotechnology, Shanghai, China). The siRNA sequences are presented in Supplementary Table S2.

### RNA isolation, reverse transcription, and real time PCR

Total RNA was isolated from cells using TRIzol (Invitrogen, Carlsbad, CA, USA) according to the manufacturer’s instructions. RNA was reverse-transcribed in cDNA using Strand cDNA Synthesis (Yeasen, Shanghai, China). Real-time PCR was performed on the cDNA using Yeasen SYBR qPCR Master Mix (Yeasen, Shanghai, China). The Bulge-Loop^Tm^ miRNA RT primer and qRT-PCR Starter Kit (Ribobio, C10712-1) was used to detect miR-486-3p expression. *GAPDH* and *U6* were used as an endogenous control for normalization. The relative fold changes in expression were analyzed using the 2^−△△CT^ method. The related primers are given in Supplementary Table S3.

### Cell proliferation and colony formation assays

The cell proliferation assay was performed using a Cell Counting Kit-8 (Yeasen, Shanghai, China). Transfected glioma cells (2 × 10^3^) were collected at 48 h post-transfection and seeded onto a 96-well plate. The OD_450_ nm was measured for 1.5 h after the addition of 10 µL CCK8 solution. For the colony-formation assay, cells were seeded onto a 6-well plate at a density of 1 × 10^3^ cells/well. After 2 weeks, the cells were fixed with 1% crystal violet stain for 20 min at room temperature. The number of visible colonies was counted using the Image J software.

### Cell migration and wound-healing assays

Cell migration assays were performed using Transwell chambers (Corning Costar, Tewksbury, MA, USA). A total of 4 × 10^4^ transfected glioma cells in 300 μL serum-free medium were seeded into the upper chamber of each insert, and 500 μL medium supplemented with 10% FBS was added to the lower chambers. After incubation at 37 °C for 24 h, cells were fixed with 1% crystal violet stain for 20 min at room temperature. For wound-healing assays, 4 × 10^5^ cells/well were seeded onto 24-well plates and cell growth was recorded at 0 h and 24 h. The cell migration rate was analyzed using the Image J software.

### Flow cytometry

The Apoptosis Detection Kit was used (Yeasen, Shanghai, China) to detect apoptosis levels in transfected cells. Cells were resuspended in 50 μL 1 × binding buffer, and 2.5 μL Annexin V-FITC or 5 μL anti-PI-PE were added to the reaction for 15 min at room temperature. Subsequently, 1 × binding buffer (300 μL) was added to each sample. Data were collected by flow cytometry (Beckman Coulter Cytoflex).

### CHIRP

The probe for CHIRP was synthesized in RiboBio, and the CHIRP experiment was carried out according to the instructions of CHIRP kit (BersinBio, Bes5104, Guangzhou, China). CHIRP experiments were conducted in accordance with BersinBio’s CHIRP Kit (China) operating guidelines, which are detailed below. (1) Cross-link Cells, digest cells with trypsin, the cell count is 1.2 × 10^8^, add 30 mL of 1 × PBS (containing 810 µL of 37% formaldehyde, formaldehyde final concentration 1%) to each tube of samples, cross-link at room temperature for 10 min with a vertical mixer; then mix with 1.375 M Glycine for 5 min; 4 °C, 1500 rpm, collect cells by centrifugation for 5 min, discard the upper layer of PBS solution, and repeat twice; (2) Cell Lysis, the sample is placed on ice, and the lysate and reselection solution in the kit are configured to perform the lysis operation on the sample. (3) Sonication, under ice bath conditions, 3 s ON, 5 s OFF, 230W power ultrasound for 20 min until the sample is clarified; (4) Preclearing, after ultrasound, the sample was added to Agarose beads, and the binding was incubated at 4 °C for 60 min on a vertical mixer; Input (protein, RNA) was reserved and 3 mL of sample was transferred, labeled as NC, odd, even, respectively. (5) Probe preparation, in which the LINC00606 probe is divided into odd and even groups. According to the operating instructions, the LINC00606 exceeds 1200 nt. The probe group is divided into two groups. The odd number is the odd group (indicating the number of the first odd probe), and the even number is the even group (indicating the number of the first even probe). Take 8 µL of odd, even and NC probe group solutions (80 pmol probe/strip/1 mL sample); After 3 min of denaturation at 85 °C, quickly transfer the ice bath. (6) Probe preparation, prepare Streptavidin Beads according to the ratio of 50 μL beads/200 pmol probes. (7) CHIRP, configure the sample-lysis hybridization system, incubate and denature at 65 °C for 10 min on a mixer, then add the probe to the denatured sample, hybridize at 37° C for 30 min, incubate and denaturate at 50 °C for 5 min, and hybridize at 37 °C for 90 min; add the CHIRP sample-probe group samples to the magnetic bead mixture respectively, and incubate and bind on a vertical mixer at room temperature for 30 min; then perform the cleaning step, the magnetic beads are collected by the magnetic frame and supernatant is removed; the samples are collected and then followed by CHIRP-RNA Isolation; CHIRP-Protein sample collection and other experiments. Details of the sequences are listed in Supplementary Table S4.

### Fluorescence in-situ hybridization (FISH)

A specific fluorescently labeled LINC00606 FISH probe was designed and synthesized by Servicebio (Wuhan, China). Fluorescence-labelled single-strand probes were hybridized. The FISH experiment was performed according to the SweAMI-FISH manufacturer’s instructions (Servicebio). In detail, we take the cell sample as an example and fix it using 4% paraformaldehyde (DEPC configuration) for 20 min, wash it with PBS three times, then digest the sample dropwise with proteinase K (20 μg/mL) for 5 min, then wash it. Treat the sample using the hybridization solution (Servicebio China) and place it in a 37 °C incubator for 1 h. Then treat the sample using the hybridization solution (containing LINC00606-FISH-probe at a concentration of 500 nM) and place it in a 40 °C hybridization oven overnight. The washing process consists of washing the SSC solution (2 × , 1 × , 0.5 ×) with different layers of concentration for 5–10 min respectively. Dilute the signal probe (1 ×) (Servicebio China) using the hybridization solution and then place it in a wet box at 40 °C for hybridization for 50 min, followed by the cleaning steps. Repeated hybridization and washing of the signal probe were performed to enhance the signal of the probe. Immunofluorescence staining was performed afterwards, and the samples were sealed and broken. Blocking was performed using 5% goat serum. The broken membrane was permeated with 1 × PBS containing 1% goat serum and 0.25% Triton ™ X-100, and then stained with ATP11B antibody overnight at 4 °C. The next day, the climbing tablets were washed 3 times with 1 × PBS for 3 min each time, incubated with fluorescent secondary antibody for 1 h at room temperature, and then washed again. DAPI (Invitrogen, NBP2311561) was used to counterstain the nuclei after incubation for 5 min in the dark. Finally, samples were washed four times with PBST for 5 min each to remove excess DAPI, and then sealed with a sealing solution containing a fluorescent quencher. All fluorescence images were captured using a laser confocal microscope (Zeiss, Germany). Details of the sequences are listed in Supplementary Table S4.

### Immunoblotting

Immunoblotting analysis was performed as described previously [26]. In detail, wash the cells with pre-cooled PBS and lyse them on ice with Western and IP lysate (Beyotime, China) for 15 min. Collect the protein lysate into an EP tube, centrifuge at 12,000 rpm for 30 min at 4 °C, collect the supernatant, and determine the soluble protein concentration using a BCA protein detection kit (Sparkjade, China). Add protein lysate to 5 × loading buffer (Beyotime, China) and boil for 10 min for SDS-PAGE (6–15%) electrophoresis, then transfer to NC or PVDF membranes. The membranes were sealed with 5% skim milk at room temperature for 2 h, incubated overnight with primary antibody at 4 °C (Abclonal, Wuhan, China), and then incubated with secondary antibody at room temperature for 1 h. Visualization of protein bands using the Tanon gel imaging system. Relative protein levels are normalized to GAPDH or Vinculin. Antibodies used in this study are shown in Supplementary Table S5.

### Dual luciferase reporter assay

Verify the relationship between LINC00606 and miR-486-3p or miR-486-3p and TCF12 using the Dual Luciferase Reporter Assay Kit (Vazyme, China). Taking miR-486-3p and TCF12 as examples, we predicted the binding sequence of miR-486-3p and TCF12 through RNA hybridization software and analyzed the free energy of its targeted binding checkpoint. We designed the mutant (MUT) binding checkpoint using the wild-type (WT) TCF12 complementary sequence to construct pGL3 Basic reporter plasmid (Tsingke Biotechnology, Shanghai, China). Transfect 293T cells with TCF12-MUT (mutant 3'UTR) or TCF12-WT (luciferase reporter plasmid, sequence correct) and miR-486-3p mimic or NC (Ribo, China) for 48 h. After the cells were lysed using the lysate in the kit, the supernatant was centrifuged at 12,000 rpm for two min for subsequent experimental detection. Luciferase substrate, cell lysate, and renilla substrate working fluid were added sequentially to the enzyme label plate, and each group was detected separately using an enzyme label instrument. Relative luciferase activity was the ratio of firefly luciferase to renal luciferase. Three independent experiments were carried out. Details of the sequences are listed in Supplementary Table S6.

### RIP

We take the cell sample as an example, wash it twice with pre-cooled PBS, then use cell scraping to collect the cells, and centrifuge to collect the cells at 4 °C to obtain the cell pellet. Add lysate (Beyotime, China) to lyse the cells, and centrifuge to take supernatant and wait for follow-up experiment. Wash the protein A/G magnetic beads (ABclonal, RM02915, Wuhan, China) with NT2 buffer (containing 50 mM Tris–HCl, 150 mM NaCl, MgCl_2_ 1 mM, 0.05% NonidetP-40, ddH_2_O), and spin the corresponding antibody ATP11B (5 μg) at room temperature for 2 h. Then collect the magnetic beads in a magnetic rack, wash and set aside. Configure the immunoprecipitation buffer (800 μL NT2 buffer, 35 μL 0.5 M EDTA, 5 μL RNase inhibitor), add the collected magnetic beads and cell lysate to the immunoprecipitation buffer and transfer to a rotator overnight at 4 °C. The reserved input group is the cell lysate. For the magnetic bead sample, perform the cleaning step. The sample is RNA purified, simply put the magnetic bead sample into the protease K solution (containing 117 μL NT2 buffer, 15 μL 10% SDS, 8 μL 10 mg/mL protease K), shake at 55 °C for 30 min, and collect the supernatant for subsequent RNA extraction experiments.

### RIP-qPCR

Protein A/G magnetic beads for 1 h (ABclonal, RM02915, Wuhan, China) were washed with NT2 buffer and incubated with anti-IgG or anti-Argonaute2 for 1 h. Finally, the magnetic bead-antibody complex was rinsed with RIP wash buffer and incubated with protein lysate overnight at 4 °C to evaluate the binding of Argonaute2 (AGO2) to LINC00606 and miR-486-3p. The magnetic bead-protein-RNA complex was reconstituted in purification solution (10 mg/mL protease K). Total RNA was obtained using TRIzol for subsequent qPCR detection. Details of the antibodies are listed in Supplementary Table S3.

### RNA pulldown

Reference RNA Labeling Mix (Roche Life Science, Germany) was used for biotin labeling of RNA, according to the manufacturer’s instructions. LINC00606 was labeled with biotin-16-UTP by in vitro transcription with T7 RNA polymerases, and RNA was purified using an RNAclean Kit DP412 (TIANGEN, Beijing, China). Biotinylated RNA was incubated with U251 lysates for 1 h at 4 °C, following which Streptavidin beads (Abclonal, RK20270, Wuhan, China) were added and further incubated overnight at 4 °C. The next day, the Streptavidin beads were washed with PBST (0.5% Tween-20), and the enriched proteins were collected for immunoblotting.

### CHIP-qPCR

The CHIP assay has been described previously [27]. U251 cells were fixed with 1% formaldehyde and collected in lysis buffer. TCF12 and IgG were used for CHIP. Immunoprecipitated DNA was amplified by PCR using specific primers (Supplementary Table S3).

### Tumor xenograft model

All animal experiments were carried out following NIH Guidelines for the Care and Use of Laboratory Animals and approved by the Animal Care Committee of Shanghai University. Cells were resuspended in 2 μL matrigel and injected into the nude mouse (Beijing Vital River Laboratory Animal Technology Co., Ltd.) brain using stereotaxic apparatus. Injection location: parietal-occipital area of mouse brain; injection coordinates 0.5 mm, -2 mm, -3 mm. Four-weeks post-injection, nude mice were sent to Huashan Hospital Affiliated to Fudan University, where the tumor marker ^18^F-fluoroethyl-tyrosine (^18^F-FET) was injected into nude mice through the tail vein and the Siemens Inveon PET/CT system was used to evaluate tumor formation in the brain. These nude mice were randomly divided into 4 groups (6 in each) for different treatments: sh-NC, sh-LINC00606, OE ATP11B, and Vector. A total of 4 × 10^6^ transfected U251 cells were injected subcutaneously into mice and growth of the resulting tumor was recorded. Mice were sacrificed after 25 days, and the volume and weight of the tumors were measured. Tumor volume was calculated as volume = length × (width)^2^/2.

### Statistical analysis

Image J, GraphPad Prism 8, Zesis, Image studio Ver 5.2, GEPIA2 (gepia2.cancer-pku.cn) were applied for analysis. The means between different groups were analyzed using a Student’s *t*-test and one-way analysis of variance. Data are expressed as the mean ± standard deviation (SD) unless otherwise noted, with a minimum of three replicates. Statistical significance was set at *P* < 0.05.

## Results

### LINC00606 is a prognostic risk factor in GBM

**Fig. 1:**
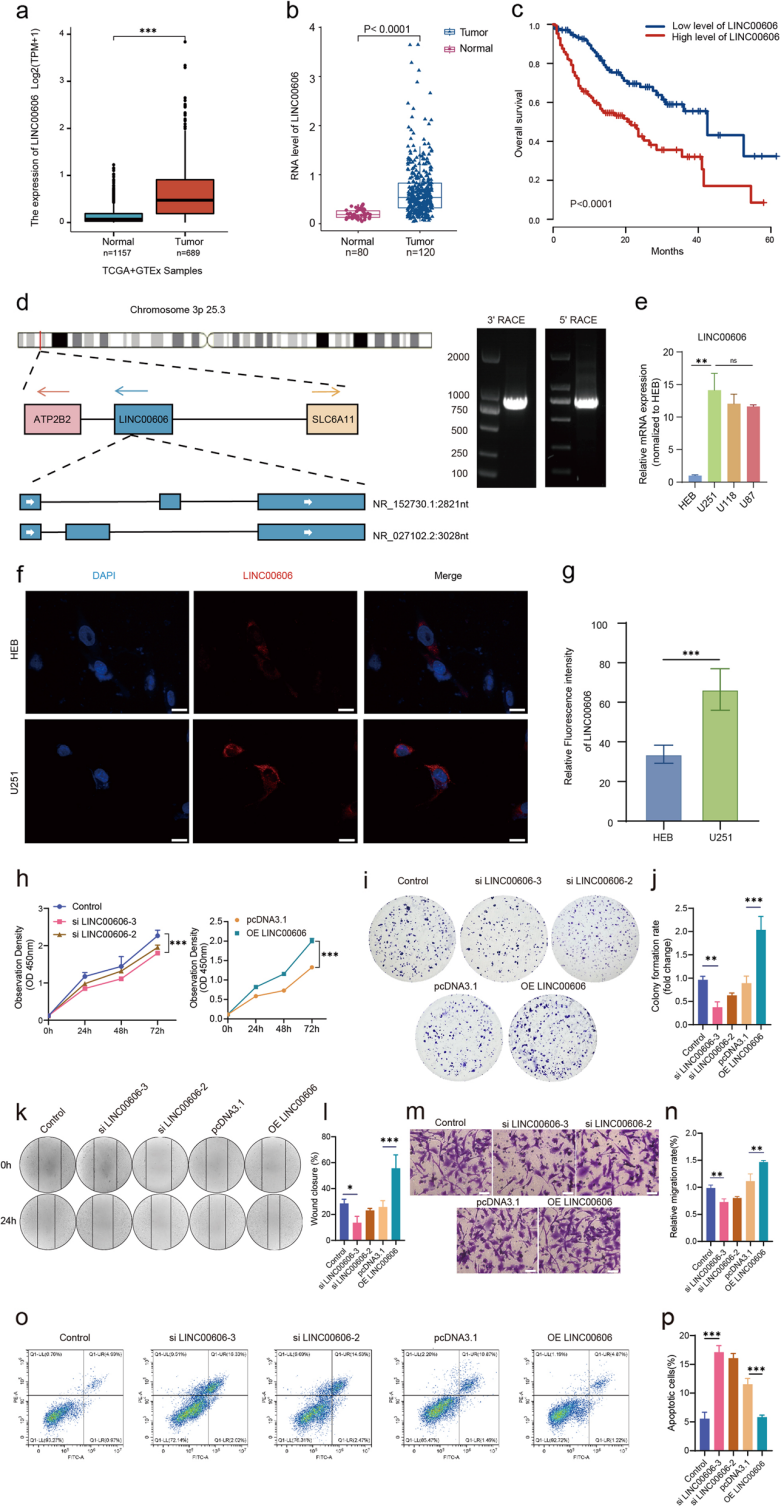
LINC00606 is a prognostic risk factor in GBM. **a** LINC00606 expression levels in glioma tissues (*n* = 689) and normal brain tissues (*n* = 1157) from the TCGA and GTEx databases. **b** QPCR was conducted to detect the expression levels of LINC00606 in glioma tissues (*n* = 120) and normal brain tissues (*n* = 80). **c** Kaplan–Meier survival curve showing the overall survival of glioma patients, grouped according to the expression level of LINC00606 (low level of LINC00606: *n* = 42; high level of LINC00606: *n* = 78). **d** LINC00606 transcript (the genomic context of LINC00606 from NCBI). Right panel: agarose gel electrophoresis of PCR products from 5’- and 3’-RACE amplification. **e** QPCR showing the mRNA expression levels of LINC00606 in U251, U118, U87, and HEB cells. **f** FISH showing the expression level of LINC00606 in U251 and HEB cells. Nuclei were stained with DAPI (blue: nuclear staining; red: LINC00606). Scale bars represent 20 μm. **g** The relative fluorescence intensity of LINC00606 in HEB and U251 cells was analyzed using the Image J software. **h** CCK-8 assay was performed to evaluate the proliferation rate of U251 cells following transfection with control siRNA (Control), LINC00606 siRNA (si LINC00606), control plasmid (pcDNA3.1), and LINC00606-overexpression plasmid (OE LINC00606). The OD value was measured at 450 nm. **i**, **j** Colony formation assay showing that LINC00606 overexpression in U251 cells promoted cell proliferation. Statistical analysis of colony numbers was performed using Image J. **k**, **l** Wound-healing assay was performed to detect the migration ability of U251 cells following transfection with si LINC00606 or OE LINC00606. Photos were taken at 0 and 24 h. A histogram was used for statistical analysis of wound-healing. **m**, **n** Transwell assay was performed to detect the migration ability of U251 cells following transfection with si LINC00606 or OE LINC00606. The number of migrated cells was analyzed using Image J. Scale bars represent 50 μm. **o**, **p** Flow cytometry was performed to analyze the apoptosis level of U251 cells following transfection with si LINC00606 and OE LINC00606. Data are expressed as the mean ± SD of three independent experiments. *P < 0.05; **P < 0.01; ***P < 0.001

To investigate the clinicopathological and prognostic significance of LINC00606 in patients with glioblastoma (GBM), the expression level of LINC00606 based on transcripts per million (TPM) in glioma tissue was measured and found to be significantly higher than that in normal brain tissue (Fig. 1a). Moreover, according to analysis of The Cancer Genome Atlas (TCGA) and Genotype-Tissue Expression (GTEx) databases, LINC00606 is specifically highly expressed in GBM and LGG tissue, and almost completely absent in other cancers (Supplementary Fig. S1a–c) [28]. To detect the expression of LINC00606 in clinical tumors, 120 glioma tissues and 80 normal brain tissue samples were collected and subjected to qPCR analysis. LINC00606 was significantly upregulated in glioma tissue in comparison with normal brain tissue (Fig. 1b). In addition, Kaplan–Meier analysis indicates that a high level of LINC00606 expression was associated with a poor prognosis in GBM patients (*P* < 0.0001, Fig. 1c). LINC00606 was localized to chromosome 3p 25.3, and full-length LINC00606 was subsequently detected by rapid 5' and 3' cDNA terminal (RACE) amplification in U251 cells (Fig. 1d). We then performed a BLAST comparison on the RACE data (NCBI website), and in comparing the two transcripts of LINC00606, our results better matched the sequence information of transcript variant 1. The full length of transcript variant 1 is 3028 nt, and the full length of transcript variant 2 is 2821 nt (Supplementary Fig. S1d, e). Moreover, the expression level of LINC00606 in U251 and U118 was significantly higher than that in the human brain glial cell line HEB. Therefore, U251 and U118 cell lines were selected for subsequent studies (Fig. 1e). Moreover, FISH results demonstrate that the expression of LINC00606 was higher in U251 cells in comparison with HEB cells (Fig. 1f, g).

To investigate the biological function of LINC00606 in glioma cells, the U251 and U118 cell lines were transfected with control siRNA (Control) or LINC00606 siRNA (si LINC00606) and with the control plasmid (pcDNA3.1) or LINC00606-overexpression plasmid (OE LINC00606) (Supplementary Fig. S2a, b). Results of CCK-8 and colony-formation assays reveal that decreased LINC00606 expression inhibited cell proliferation (Fig. 1h; Supplementary Fig. S2 c) and colony formation (Fig. 1i, j; Supplementary Fig. S2d, e) of U251 and U118 cells. Moreover, overexpression of LINC00606 yielded the opposite results, promoting cell proliferation and colony formation. Wound healing and migration assays confirm these results, since LINC00606 overexpression improved cell migration and the wound healing rate (Fig. 1k-n; Supplementary Fig. S2f-i), while LINC00606 knockdown exerted the opposite effects. Furthermore, Annexin V/PI flow cytometry demonstrates that the apoptosis rate of LINC00606-overexpressing U251 cells was significantly lower in comparison with the Control, while LINC00606-knockdown U251 cells exhibited the opposite results (Fig. 1o, p).

### LINC00606 acts as a molecular sponge for miR-486-3p in U251 cells

**Fig. 2:**
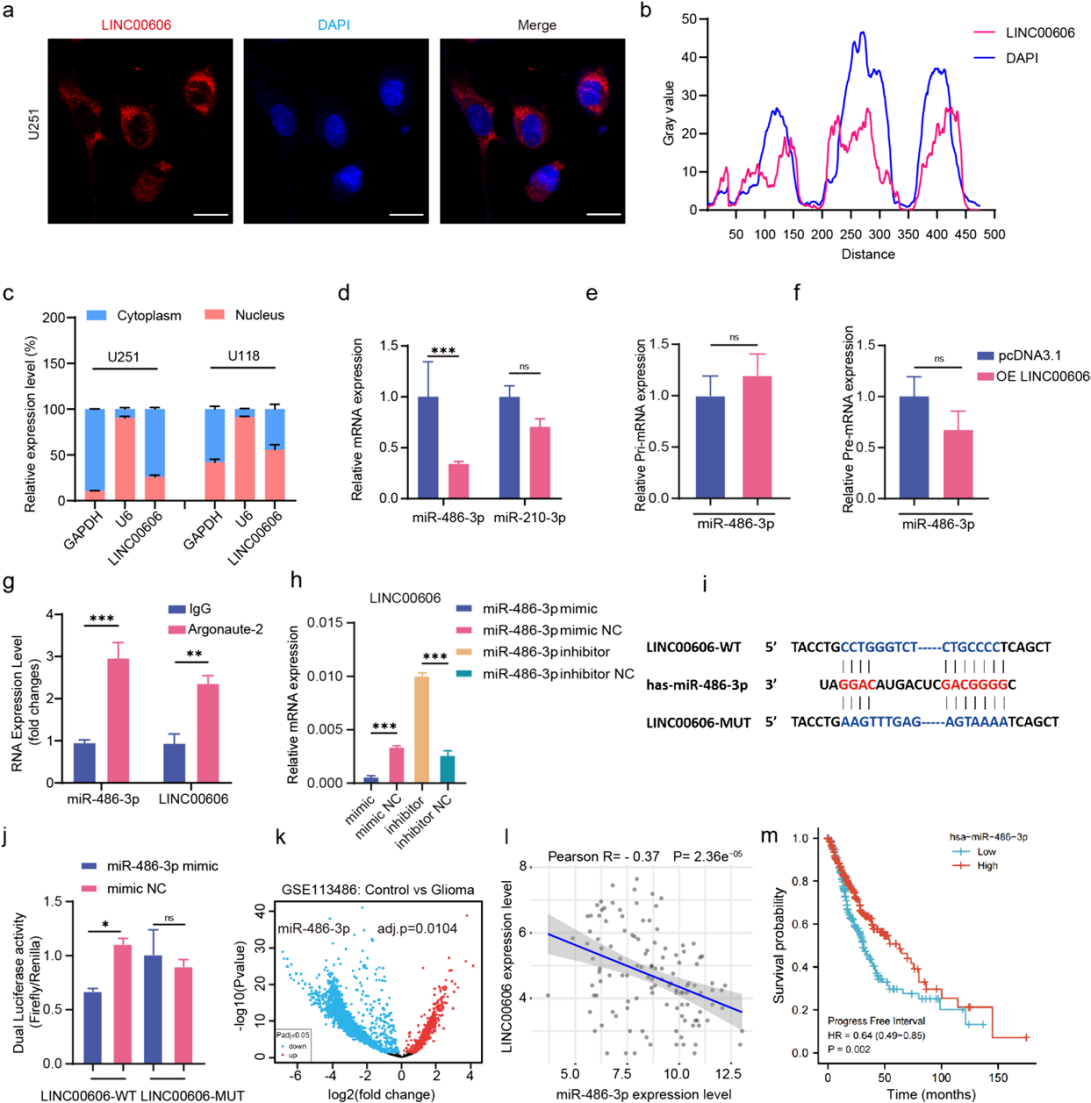
LINC00606 acts as a molecular sponge for miR-486-3p in U251 cells. **a** LINC00606 detection in U251 cells using fluorescence in-situ hybridization (FISH) probes (blue: DAPI nuclear staining; red: LINC00606). Scale bars represent 20 μm. **b** Gray FISH values for LINC00606 and DAPI in U251 cells. Red represents LINC00606; blue represents DAPI. **c** RNA samples were collected from the cytoplasm and nucleus of U251 and U118 cells, and the proportion of LINC00606 mRNA was analyzed. **d**-**f** Expression levels of miR-486-3p, miR-210-3p, pri-miR-486-3p and pre-miR-486-3p in U251 cells transfected with OE LINC00606 and pcDNA3.1. **g** RIP assay was performed using rabbit anti-AGO2 and IgG antibodies in U251 cells. Relative expression levels of LINC00606 and miR-486-3p were determined by qPCR. **h** Relative mRNA expression of LINC00606 in U251 cells transfected with the miR-486-3p mimic, mimic NC, inhibitor, and inhibitor NC. **i** According to the predicted binding site between LINC00606 and miR-486-3p, LINC00606 wild-type (LINC00606-WT) and LINC00606 mutant (LINC00606-MUT) plasmids were constructed. **j** Dual luciferase activity in HEK293T cells co-transfected with the miR-486-3p mimic and LINC00606-WT or LINC00606-MUT. **k** MiR-486-3p expression levels in non-cancer control (*n* = 100) and glioma (*n* = 40) patients from the GSE113486 database. **l** Correlation analysis between the expression levels of LINC00606 and miR-486-3p in TCGA and CGGA databases (GBM clinical samples, *n* = 121). **m** Kaplan–Meier survival curve shows the overall survival of glioma patients, grouped according to the expression level of miR-486-3p. Data are expressed as the mean ± SD of three independent experiments. *P < 0.05; **P < 0.01; ***P < 0.001

To further explore the molecular mechanism of LINC00606 in glioma progression, we determined its subcellular localization. FISH results show that the majority of LINC00606 was located in the cytoplasm of U251 cells (Fig. 2a, b). QPCR performed using the cytoplasmic and nuclear fractions of U251 and U118 cells demonstrates that LINC00606 was mainly distributed in the cytoplasm of U251 cells; however, almost equal expression was found in the cytoplasm and nucleus of U118 cells (Fig. 2c). Since lncRNAs can function as competing endogenous RNA that typically bind their target miRNA through complementary base pairing to regulate target gene expression [29], we used the DIANA Tools (https://diana.e-ce.uth.gr/lncbasev3/interactions) database to find target miRNA for LINC00606. The results indicate that miR-210-3p and miR-486-3p may bind to LINC00606 (Supplementary Fig. S3a); Therefore, we analyzed the expression levels of these miRNAs in U251 cells following overexpression of LINC00606. The expression level of miR-210-3p remained unchanged in the OE LINC00606 group, whereas miR-486-3p was downregulated (Fig. 2d). Therefore, miR-486-3p was selected for further investigation. We first observed higher levels of miR-486-3p expression in HEB cells compared to U251 and U118 cells (Supplementary Fig. S3b). To evaluate whether LINC00606 regulates the primary transcription of pri-miR-486-3p and pre-miR-486-3p, we overexpressed LINC00606 and assessed the levels of pri-miR-486-3p and pre-miR-486-3p in U251 cells by qPCR. The results showed that overexpression LINC00606 did not alter the expression levels of pri-miR-486-3p or pre-miR-486-3p in U251 cells (Fig. 2e, f). In addition, RNA immunoprecipitation (RIP) shows that AGO2 was bound to LINC00606 and miR-486-3p (Fig. 2g). These results indicate that LINC00606 regulates the expression of miR-486-3p in U251 cells at the post-transcriptional level.

Subsequently, we measured the mRNA expression level of LINC00606 in U251 cells following transfection with the miR-486-3p mimic, mimic NC, miR-486-3p inhibitor, and inhibitor NC. The mRNA expression level of LINC00606 was significantly reduced in cells transfected with the miR-486-3p mimic, while it was upregulated in cells transfected with the miR-486-3p inhibitor (Fig. 2h). These results indicate that LINC00606 sponged miR-486-3p to reduce its expression in U251 cells. To further verify binding between miR-486-3p and LINC00606, we predicted their binding site and designed wild-type (LINC00606-WT) and mutant (LINC00606-MUT) plasmids (Fig. 2i) for dual luciferase reporter assays. miR-486-3p significantly repressed the luciferase activity of LINC00606-WT in comparison with mimic-NC in U251 cells, while miR-486-3p did not affect the luciferase activity of LINC00606-MUT (Fig. 2j). These results confirm that LINC00606 sponged miR-486-3p to regulate its expression in U251 cells.

To explore the potential of miR-486-3p for the intervention in glioma progression, we evaluated its expression and found it to be low, which was confirmed by GSE113486 data (Fig. 2k). In addition, we evaluated TCGA and CGGA databases (GBM clinical samples, *n* = 121), and correlation analysis showed that LINC00606 and miR-486-3p expression levels were inversely correlated (Fig. 2l). Finally, for the purpose of verifying the prognostic value of miR-486-3p in glioma, we analyzed the survival probability of 169 glioma patients (high = 85, low = 84) from the TCGA database by Kaplan Meier, which demonstrates that a high expression of miR-486-3p was related to better prognosis in glioma patients (Fig. 2m).

### The tumor suppressor functions of miR-486-3p and its target gene *TCF12* regulate the transcription of LINC00606, PTEN and KLLN

**Fig. 3:**
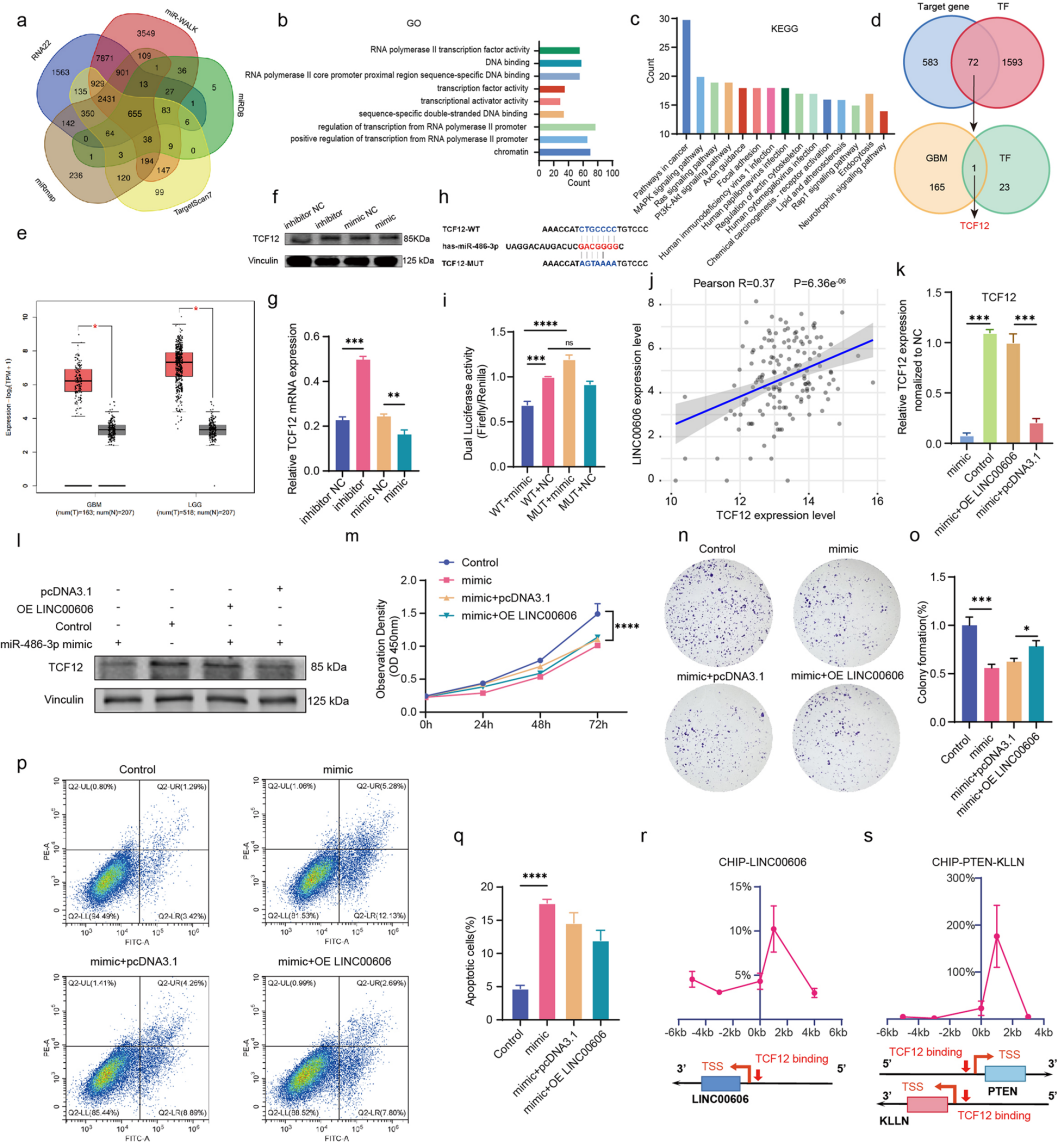
The tumor suppressor functions of miR-486-3p and its target gene TCF12 regulate the transcription of LINC00606, PTEN, and KLLN. **a** Venn diagram showing the predicted target genes of miR-486-3p using the RNA22, miR-WALK, miRDB, miRmap, and TargetScan7 databases. **b**, **c** Gene Ontology (GO) and Kyoto Encyclopedia of Genes and Genomes (KEGG) functional enrichment analysis of the 655 target genes. **d** Top: Venn diagram showing the 655 target genes and transcription factors, with the 72 overlapping genes. Bottom: Venn diagram showing the intersection of transcription factors and DEGs in GBM within the TCGA and GTEx databases. **e** The TCGA and GTEx databases were used to analyze the expression of *TCF12* in GBM (*n* = 163) vs normal (*n* = 207) tissue samples and in LGG (*n* = 518) vs normal (*n* = 207) tissue samples. **f** Immunoblotting was used to measure the protein expression level of TCF12 in U251 cells transfected with the miR-486-3p mimic, mimic NC, miR-486-3p inhibitor, and inhibitor NC. **g** QPCR was performed to evaluate the mRNA expression of TCF12 in U251 cells transfected with the miR-486-3p mimic, mimic NC, miR-486-3p inhibitor, and inhibitor NC. **h** According to the binding site between *TCF12* and miR-486-3p, wild-type TCF12 (TCF12-WT) and mutant TCF12 (TCF12-MUT) plasmids were constructed. **i** Dual luciferase activity in HEK-293 T cells co-transfected with TCF12 wild-type or mutant and the miR-486-3p mimic or mimic NC. **j** Correlation analysis between the expression levels of TCF12 and LINC00606 in TCGA data (GBM clinical samples, *n* = 145). **k** Relative mRNA expression of TCF12 in U251 cells transfected with the miR-486-3p mimic, miR-486-3p NC (Control), miR-486-3p mimic + pcDNA3.1, or OE LINC00606 + pcDNA3.1 as measured by qPCR. **l** Protein expression of TCF12 in U251 cells transfected with the miR-486-3p mimic, miR-486-3p NC (Control), miR-486-3p mimic + pcDNA3.1, or OE LINC00606 + pcDNA3.1 as measured by immunoblotting. **m** The CCK-8 assay was applied to evaluate the proliferation of U251 cells transfected with the miR-486-3p mimic, miR-486-3p NC (Control), miR-486-3p mimic + pcDNA3.1, or OE LINC00606 + pcDNA3.1. **n**, **o** Colony formation assay of U251 cells showing the proliferation activity following transfection with the miR-486-3p mimic, miR-486-3p NC (Control), miR-486-3p mimic + pcDNA3.1, or OE LINC00606 + pcDNA3.1. Statistical analysis of colony number was performed using Image J. **p**, **q** Flow cytometry of the apoptotic rate of U251 cells transfected with the miR-486-3p mimic, miR-486-3p NC (Control), miR-486-3p mimic + pcDNA3.1, or OE LINC00606 + pcDNA3.1. **r** Association of TCF12 with the promoter region of LINC00606 in U251 cells as shown by CHIP-qPCR. **s** Association of TCF12 with the promoter regions of PTEN and KLLN in U251 cells as shown by CHIP-qPCR. Data are expressed as the mean ± SD of three independent experiments. *P < 0.05; **P < 0.01; ***P < 0.001; ****P < 0.0001

Previous studies have shown that miR-486-3p is a tumor suppressor [30, 31] and high expression of miR-486-3p may also enhance the sensitivity of chemotherapeutic drugs in the treatment of GBM [32]; however, its biological role and specific mechanism in GBM remains to be elucidated. To investigate the mechanism underlying miR-486-3p function in glioma, we continued to explore its target genes using the RNA22, miR-WALK, miRDB, miRmap, and TargetScan7 databases, and a total of 655 genes were found to appear across all five databases (Fig. 3a). Subsequently, Gene Ontology (GO) and Kyoto Encyclopedia of Genes and Genomes (KEGG) functional enrichment analysis was performed on these 655 genes to further explore the biological function of miR-486-3p. GO results show that the target genes were mainly enriched in chromatin and transcriptional initiation (Fig. 3b), while KEGG results reveal that miR-486-3p may be mainly involved in cancer pathways and PI3K/AKT signaling pathway (Fig. 3c), suggesting that miR-486-3p may have epigenetic regulatory potential in tumor progression.

Transcription factors (TFs) play a critical role in tumorigenesis, tumor progression, and drug response [33]; therefore, we further screened TFs for the 655 target genes and found a total of 72 (Fig. 3d). To assess whether any of these TFs have regulatory significance in GBM, we compared their expression between glioma and normal brain tissues from the TCGA and GTEx database using the GEPIA2 website (http://gepia2.cancer-pku.cn/#index) (Supplementary Fig. S4); and interestingly, 24 TFs were differentially expressed. Encouragingly, screening of the differentially expressed genes (DEGs) in GBM within the TCGA and GTEx databases using the Top10 method uncovered only *TCF12* enriched at the intersection of DEGs and TFs (Fig. 3d); therefore, we chose *TCF12* as the target gene of miR-486-3p for further study. Moreover, *TCF12* was highly expressed in LGG and GBM (Fig. 3e), as well as in four different subtypes of GBM (Supplementary Fig. S5a). Furthermore, TCF12 displayed brain tissue-specific expression, and pan-cancer enrichment analysis also shows that TCF12 expression in LGG and GBM was high (Supplementary Fig. S5b, c). In addition, we detected the expression level of TCF12 in HEB, U251, and U118 cells (Supplementary Fig. S5d), the expression level of TCF12 is significantly increased in glioma cell lines. To verify the influence of miR-486-3p on the expression of TCF12, we evaluated the mRNA and protein expression levels of TCF12 following transfection with the miR-486-3p mimic, mimic NC, miR-486-3p inhibitor, and inhibitor NC. We found that both the mRNA and protein expression levels of TCF12 were significantly reduced in U251 cells transfected with the miR-486-3p mimic, while these were upregulated following transfection with the inhibitor (Fig. 3f, g). A luciferase reporter experiment was carried out to confirm that *TCF12* is a target gene of miR-486-3p. Transfection of TCF12-WT-expressing U251 cells with miR-486-3p reduced the luciferase activity in comparison with TCF12-MUT-expressing U251 cells (Fig. 3h, i), confirming binding between miR-486-3p and *TCF12*. In addition, we evaluated TCGA data (GBM clinical samples, *n* = 145), and correlation analysis showed that LINC00606 and TCF12 expression levels were positively correlated (Fig. 3j). These data confirm that *TCF12* is the direct target gene of miR-486-3p sponged by LINC00606.

Based on our results, we speculated that miR-486-3p may target *TCF12* and mediate the malignant progression of glioma; therefore, an in vitro rescue experiment was conducted to evaluate the regulatory role of LINC00606 via its direct sponging of miR-486-3p and subsequent influence on *TCF12*. Firstly, to demonstrate the effect of LINC00606 on the regulation of the miR-486-3p target gene *TCF12*, we transfected U251 cells with the miR-486-3p mimic, and the mRNA and protein expression levels of TCF12 decreased significantly. We then co-transfected U251 cells with the miR-486-3p mimic and the LINC00606-overexpression plasmid, which rescued the mRNA and protein expression levels of TCF12 (Fig. 3k, l). Previous experimental results showed that overexpression of LINC006006 can significantly improve the proliferation and colony formation abilities and reduce the apoptotic rate of U251 cells. We further confirmed that miR-486-3p is involved in regulating the biological function of U251 cells, since increased expression of miR-486-3p significantly decreased the proliferation and colony formation abilities and significantly increased the level of apoptosis (Fig. 3m–q). It is noteworthy that the apoptosis level of U251 cells co-transfected with the miR-486-3p mimic and LINC00606-overexpression plasmid was lower than that of U251 cells transfected with the miR-486-3p mimic alone (Fig. 3p, q), indicating that miR-486-3p increased glioma cell apoptosis but overexpression of LINC00606 antagonized this apoptosis-promoting effect. Previous study reported that TCF12 enhances cell proliferation, migration, and invasion of hepatocellular carcinoma by activating the PI3K/AKT signaling pathway [34]. As a critical regulatory factor in the PI3K/AKT signaling pathway, PTEN regulates the Phosphoinositide 3-kinase (PI3K) and Protein Kinase B (AKT) pathway, which is involved in the cell proliferation and apoptosis of various cancers [35, 36]. GO terms suggest that TCF12 has DNA-binding and transcription factor activity (Fig. 3b); therefore, we speculate that TCF12 regulates tumor malignant progression by mediating gene transcription. We subsequently predicted the key genes and corresponding promoter binding sites in LINC00606 and the PI3K signaling pathway according to the sequence of TCF12. CHIP-qPCR demonstrates that TCF12 bound to the promoter region of LINC00606 in U251 cells (Fig. 3r). Interestingly, TCF12 also bound to the promoters of *PTEN* and *KLLN* to initiate two-way transcription of these genes (Fig. 3s). PTEN is a tumor suppressor downstream of the PI3K signaling pathway, while KLLN inhibits DNA synthesis and induces apoptosis [37, 38]. We tried to overexpress PTEN and KLLN in U251 and U118 cells, and then used the apoptosis detection kit and flow cytometry to perform statistical analysis of cell apoptosis rate. The results showed that the apoptosis levels of U251 and U118 cells increased after overexpressing PTEN or KLLN (Supplementary Fig. S6a, b). In addition, CCK8 results showed that overexpression of PTEN or KLLN in U251 and U118 cells resulted in a decrease in cell proliferation, suggesting that PTEN and KLLN also affect glioma cell viability (Supplementary Fig. S6c, d). PTEN and KLLN can participate in the inhibition of tumor development and the regulation of cancer progression. In summary, miR-486-3p can alleviate the carcinogenic effect of LINC00606, and the target gene *TCF12* can regulate the transcriptional initiation of LINC00606, PTEN, and KLLN.

### LINC00606 regulates carcinogenesis by cooperating with ATP11B in U251 cells

**Fig. 4:**
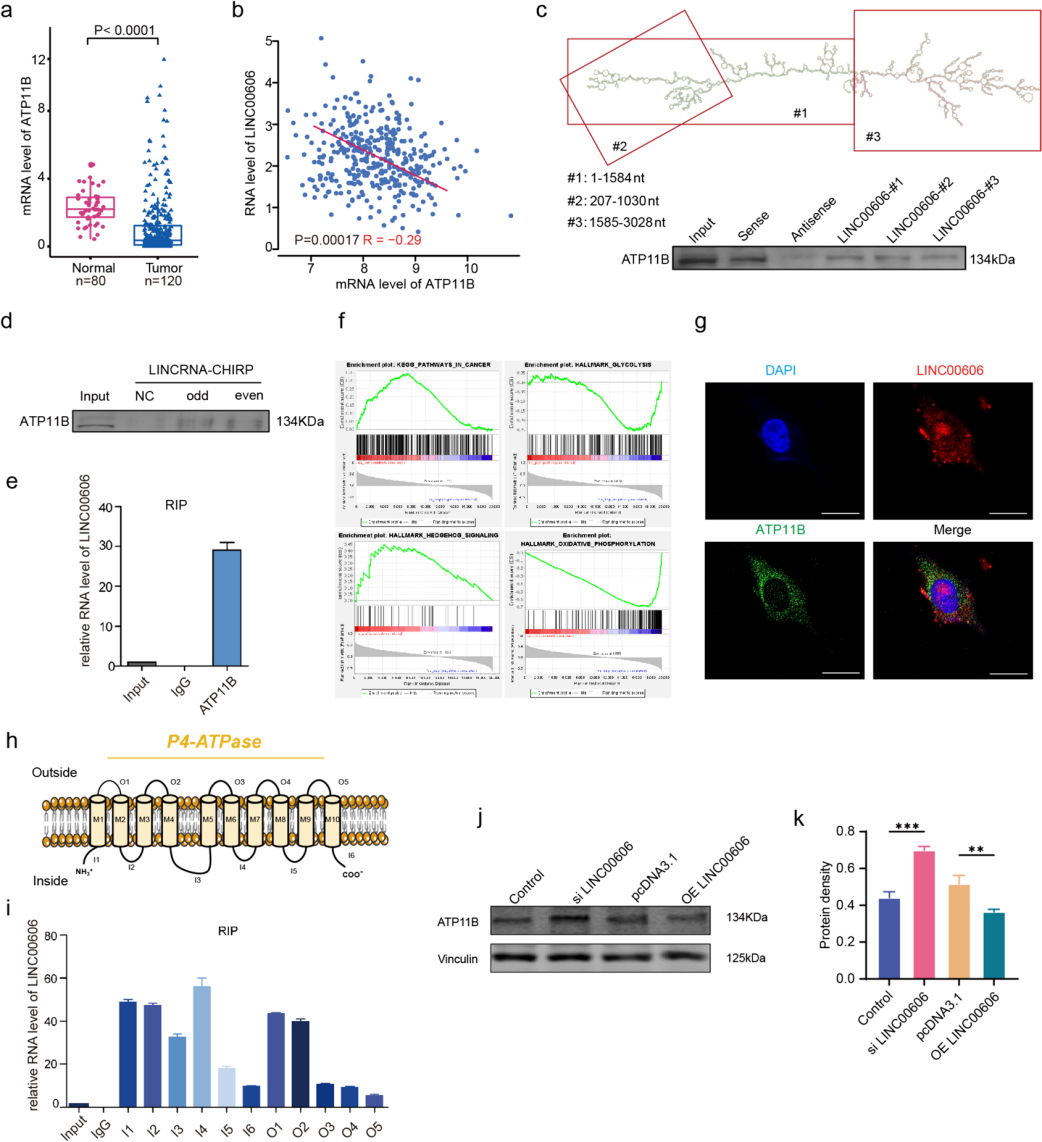
LINC00606 negatively regulate ATP11B. **a** ATP11B mRNA expression levels in glioma (tumor: *n* = 120) and normal brain (normal: *n* = 80) tissues. **b** Correlation analysis between ATP11B and LINC00606 in glioma (*n* = 120) and normal brain (*n* = 80) tissues. **c** The secondary structure of LINC00606 was divided into three parts: #1: 1–1584nt, #2: 207–1030nt, and #3: 1585–3028nt. The secondary structure was obtained from the RNAfold web server. RNA pulldown showing that 1–1584nt of LINC00606 is the main structural region interacting with ATP11B. **d** CHIRP immunoblotting showing that ATP11B interacts with LINC00606. **e** The interaction between LINC00606 and ATP11B was detected using RIP assays in U251 cells. **f** GSEA enrichment analysis based on RIP-seq data. **g** Colocalization of ATP11B (green) and LINC00606 (red) in U251 cells as shown by fluorescence microscopy. DAPI staining (blue) shows the nuclei (DNA). Scale bars represent 20 μm. **h** Structural diagram of ATP11B. O: outside the membrane, I: inside the membrane. **i** RIP assay showing the interaction between LINC00606 and each segment of the transmembrane domain (including 6 intracellular segments and 5 extracellular segments) of ATP11B. **j**, **k** Protein expression level of ATP11B in U251 cells transfected with Control, si LINC00606, pcDNA3.1 or OE LINC00606. Data are expressed as the mean ± SD of three independent experiments. *P < 0.05; **P < 0.01; ***P < 0.001

LncRNAs typically bind directly to proteins to regulate the occurrence and development of disease [39]. In order to further uncover the regulatory mechanism of LINC00606 in GBM, we decided to search for functional proteins that bind to it, for which we synthesized biotin-labeled LINC00606 probes in vitro. RNA pull-down and mass spectrometry were performed to explore potential protein binding partners, and we screened 18 bindings proteins (unique peptide number > 8, PG coverage > 25%) as candidates for LINC00606 to exert tumor regulatory mechanisms in the GBM (Supplementary Table S7). Previous studies have shown that the candidate protein ATP11B is an inhibitor of cancer metastasis [40] and displays heterogeneous expression in various nerve cell-s in the brain [24]. ATP11B is closely related to the field of neurology or oncology, so we selected ATP11B as the binding protein for our research. Analysis of the 200 clinical samples demonstrates that the mRNA expression level of ATP11B in glioma tissues was significantly reduced in comparison with that in normal brain tissues (Fig. 4a). Kaplan–Meier analysis of the overall survival indicates that higher ATP11B expression was associated with a better prognosis in GBM patients (Supplementary Fig. S7a). In addition, the expression of ATP11B was negatively correlated with that of LINC00606 in GBM patient’s tumor samples (Fig. 4b). Wilcoxon rank-sum analysis of differential lncRNAs in the RIP-seq data from ATP11B-overexpressing U251 cells reveals that LINC00606 had a more significant enrichment difference in the brain than other long non-coding RNA (Supplementary Fig. S7b). We used the catRAPID website to predict the binding relationship between LINC00606 and ATP11B, uncovering that LINC00606 and ATP11B possess RNA binding domains (Supplementary Fig. S7c–e). Next, a truncated plasmid was constructed according to the secondary structure of LINC00606 obtained from the RNAfold web server. RNA pulldown demonstrates that the 1–1584 nt region of LINC00606 was the main RNA-binding domain interacting with ATP11B (Fig. 4c; Supplementary Fig. S7f). To explore whether LINC00606 functions in the cytoplasm by binding to ATP11B, we synthesized a specific LINC00606 probe for Chromatin Isolation by RNA Purification (CHIRP). Odd and even CHIRP probe group LINC00606, which compared to the NC control group, has a binding relationship with ATP11B, which is reflected in the protein band signal and CHIRP-qPCR data in Fig. 4d, Supplementary Fig. S7g. RIP experiments confirmed the relationship between LINC00606 and ATP11B (Fig. 4e). It is noteworthy that GSEA was enriched in pathways associated with cancer, such as those involving pathways in cancer, and oxidative phosphorylation, hedgehog signaling, as well as a development of glycolysis (Fig. 4f).

To provide additional evidence for the interaction between ATP11B and LINC00606, we first determined that they co-localize based on the FISH fluorescence staining results (Fig. 4g, Supplementary Fig. S7h). To further verify the binding domains between ATP11B and LINC00606, we divided ATP11B into five extracellular segments and six intracellular segments according to the transmembrane sequence (https://web.expasy.org/translate/) (Fig. 4h), which were confirmed by gel electrophoresis (Supplementary Fig. S7g). Subsequently, we constructed an expression plasmid for each ATP11B segment and carried out RIP experiments. All segments of ATP11B bound to LIN00606 to some extent; however, the four intracellular segments (I1–I4: within the membrane) displayed stronger binding (Fig. 4i). When we transfected si LINC00606 to reduce cell expression levels, the results showed no significant changes in ATP11B expression, suggesting that LINC00606 did not affect ATP11B expression at RNA level (Supplementary Fig. S7j, k). Finally, immunoblotting shows that the ATP11B expression level was significantly decreased in U251 cells following transfection with the LINC00606-overexpression plasmid, while the expression level of ATP11B in U251 cells transfected with LINC00606 siRNA was increased (Fig. 4j, k). These results indicate that LINC00606 interacted with ATP11B and inhibited its protein expression level.

### LINC00606 and ATP11B affect the biological behavior of glioma cells and regulate apoptosis by participating in the PI3K/AKT signaling pathway

**Fig. 5:**
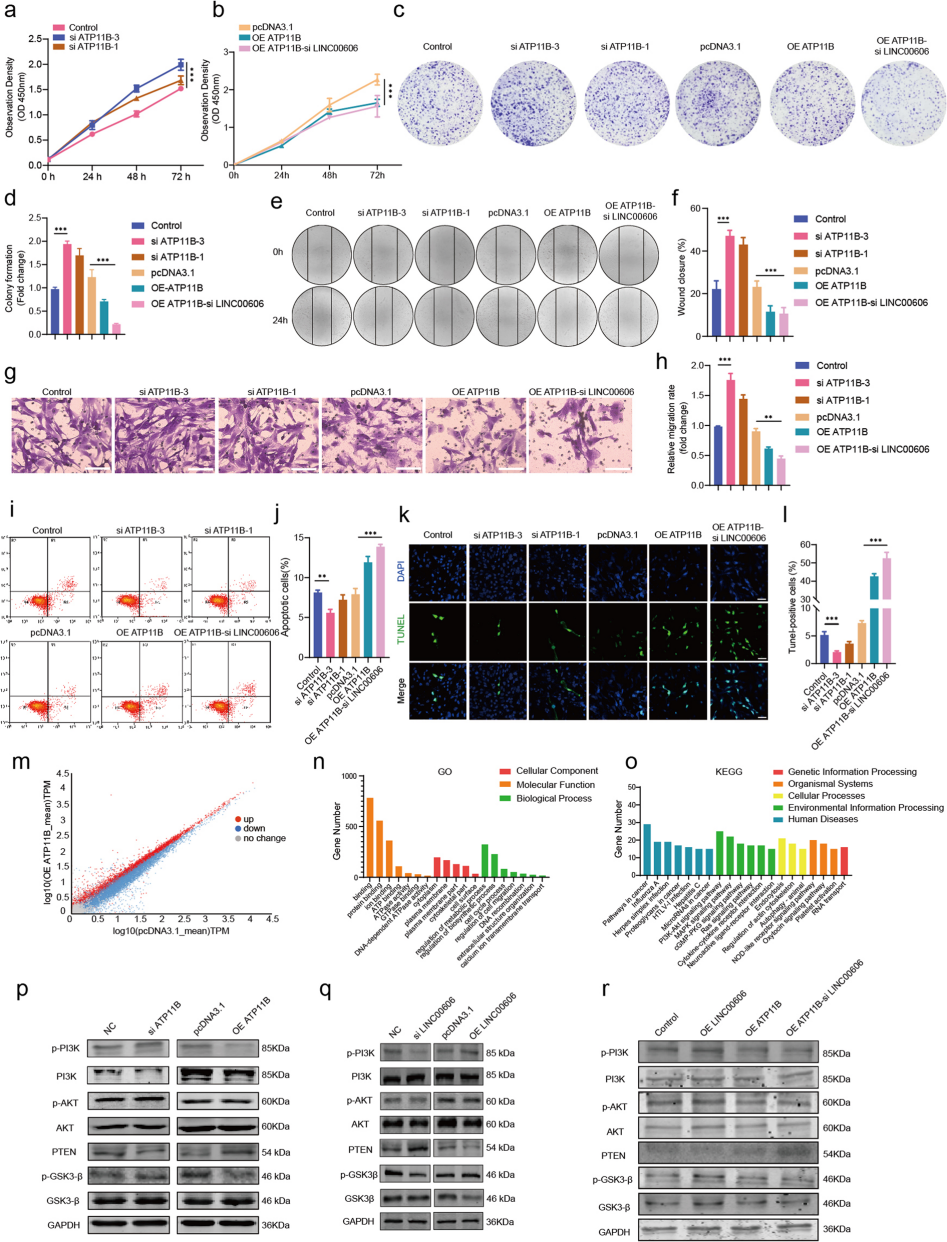
LINC00606 and ATP11B affect the biological behavior of glioma cells and regulate apoptosis by participating in the PI3K/AKT signaling pathway. **a** CCK-8 assay was applied to evaluate the proliferation rate following transfection of U251 cells with control siRNA (Control) or ATP11B siRNA (si ATP11B). **b** CCK-8 assay was applied to evaluate the proliferation rate of U251 cells following transfection with the control plasmid (pcDNA3.1) or the ATP11B-overexpression plasmid (OE ATP11B) or co-transfection with the ATP11B-overexpression plasmid and LINC00606 siRNA (OE ATP11B-si LINC00606). **c, d** Colony formation assay of U251 cells showing that knockdown of ATP11B promotes cell proliferation. Statistical analysis of colony number was performed using Image J. **e**, **f** Wound-healing assay was used to detect the migration ability of U251 cells with or without si ATP11B and OE ATP11B. Photos were taken at 0 and 24 h. A histogram was used for statistical analysis of wound-healing. **g**, **h** Transwell assay was used to detect the migration ability of U251 cells with or without si ATP11B and OE ATP11B. The number of migrated cells was analyzed using Image J. Scale bars represent 100 μm. **i**, **j** Flow cytometry was performed to analyze the changes in apoptosis level of U251 cells with or without si ATP11B, si LINC00606 and OE ATP11B. **k**, **l** A higher percentage of U251 cells overexpressing ATP11B are TUNEL-positive (green). Statistical analysis of TUNEL-positive cells (green) was performed and the ratio of apoptotic cells was analyzed using Image J. Scale bars represent 20 μm. **m** Scatter plot of upregulated (red) and downregulated (blue) genes in Control and ATP11B-overexpressing U251 cells. **n**, **o** GO and KEGG enrichment analyses were performed on 1630 DEGs. **p**–**r** Immunoblotting showing the relative expression levels of p-PI3K, PI3K, p-AKT, AKT, PTEN, GSK3β, and p-GSK3β regulated by ATP11B and LINC00606 in U251 cells. GAPDH were used as internal controls. Data are expressed as the mean ± SD of three independent experiments. *P < 0.05; **P < 0.01; ***P < 0.001

To further explore the role of ATP11B in the proliferation and migration of glioma cells, we knocked down ATP11B or overexpressed ATP11B in U251 and U118 cells (Supplementary Fig. S8a, b). According to the CCK-8 results, knockdown of ATP11B upregulated the proliferation of U251 and U118 cells; however, proliferation was significantly inhibited in U251 cells overexpressing ATP11B and LINC00606 siRNA (Fig. 5a, b; Supplementary Fig. S8c, d). Knockdown of ATP11B improved the colony formation ability of U251 and U118 cells (Fig. 5c, d; Supplementary Fig. S8e, f). Furthermore, ATP11B knockdown increased the migration ability of U251 and U118 cells as shown by Wound-healing and Transwell assays (Fig. 5e–h; Supplementary Fig. S8g–j). In addition, flow cytometry and TUNEL assays show that overexpression of ATP11B significantly increased the apoptosis of glioma cells (Fig. 5i–l; Supplementary Fig. S8k, l).

Next, we evaluated the differentially expressed genes in U251 cells transfected with pcDNA3.1 or OE ATP11B by transcriptome sequencing (RNA-seq) and then screened the 1630 DEGs (Fig. 5m). GO enrichment (Fig. 5n) shows that the DEGs were mainly enriched in binding, cytoplasm, and regulation of metabolic processes. KEGG enrichment analysis yielded very similar results to those of miR-486-3p target genes (Fig. 5o), which were mainly enriched in cancer and PI3K/AKT signaling pathways, suggesting that ATP11B may modulate the PI3K/AKT signaling pathway to affect the occurrence and development of glioma. To investigate the effect of ATP11B on the PI3K/AKT signaling pathway in glioma, U251 cells were transfected with OE ATP11B or siATP11B, and the protein expression levels of p-PI3K, PI3K, p-AKT, AKT, PTEN, p-GSK3β, and GSK3β were investigated by immunoblotting (Fig. 5p; Supplementary Fig. S9a–d). Statistical analysis shows that in comparison with the Control, the expression levels of p-PI3K/PI3K, p-AKT/AKT, and p-GSK3β/GSK3β in U251 cells were significantly reduced following overexpression of ATP11B, and the expression level of PTEN (as a negative regulator of tumor suppressors and PI3K/AKT) was significantly increased. Moreover, in ATP11B-knockdown U251 cells, the protein expression level of PTEN was decreased (Fig. 5p). Interestingly, research into the effect of LINC00606 on the PI3K/AKT signaling pathway uncovered that the protein expression levels in U251 cells were different from those in U251 cells overexpressing ATP11B. Statistical analysis shows that in comparison with the control, the expression levels of p-PI3K/PI3K, p-AKT/AKT, and p-GSK3β/GSK3β in U251 cells were improved following overexpression of LINC00606. Moreover, the expression of PTEN was downregulated in U251 cells overexpressing LINC00606 and upregulated following simultaneous overexpression of ATP11B and knockdown of LINC00606 (Fig. 5q, r; Supplementary Fig. S9e–l). As expected, the interaction between LINC00606 and ATP11B is involved in the control of the biological behavior of glioma cells and participates in the regulation of the PI3K/AKT signaling pathway to modulate the apoptosis rate.

### Knockdown of LINC00606 or overexpression of ATP11B inhibits GBM progression in vivo

**Fig. 6:**
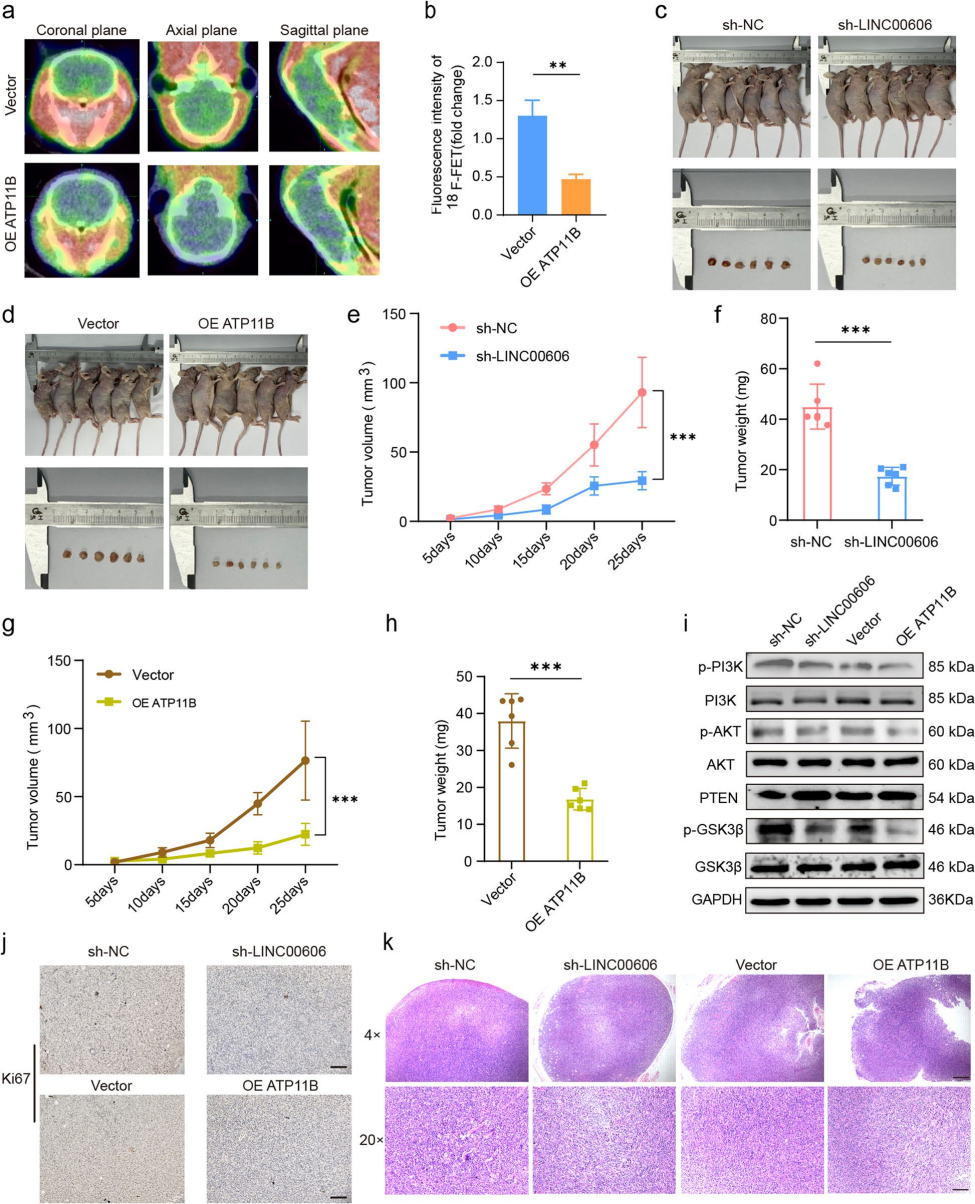
Evaluation of the effects of LINC00606 and ATP11B on glioma tumorigenesis. **a** ^18^F-FET PET images of the mouse brain showing the expression level of L-type acid amino transporter (LAT) (green), which was used to reflect the occurrence of intracranial tumors in mice. **b** Quantitative analysis of the fluorescence intensity of ^18^F-FET in the mice brain injected with U251 cells expressing vector or OE ATP11B. **c**-**d** The subcutaneous tumor model; sizes of the xenografted tumor samples were recorded by photography. **e**–**h** Weights and volumes of the xenograft tumors derived from the OE-ATP11B, Vector, sh-NC and sh-LINC00606 Lentiviral transfected U251 cells. Tumor volumes were calculated as: volume = length × (width).^2^/2. Data are expressed as the mean ± SD (each group: *n* = 6). **i** Immunoblotting showing the relative expression levels of p-PI3K, PI3K, p-AKT, AKT, PTEN, GSK3β, and p-GSK3β regulated by ATP11B and LINC00606 in U251 cells. GAPDH were used as internal controls. **j** IHC showing the expression of Ki67 in the tumor sections. Scale bars represent 100 μm. **k** H&E-stained tumor tissue sections from the OE-ATP11B, vector, sh-NC and sh-LINC00606 treatment groups. Scale bars represent 100 μm (20 ×), 200 μm (4 ×). Data are expressed as the mean ± SD of three independent experiments. *P < 0.05; **P < 0.01; ***P < 0.001

We elucidated the interaction between LINC00606 and ATP11B and the mechanism by which ATP11B, as an interacting protein, inhibits glioma cell proliferation, migration, and colony formation and increases the rate of apoptosis. Next, to explore the effect of ATP11B in vivo, U251 cells overexpressing ATP11B (OE ATP11B) was intracranially injected into nude mice in order to construct an in vivo model of glioma. PET technology is used in neuro-oncology to diagnose brain tumors, evaluate prognosis, perform image-guided surgery, and monitor tumor response after chemotherapy or radiation therapy [41]; therefore, ^18^F-FET is commonly performed to diagnose glioma [42]. One month post-creation of the model, the expression of ^18^F-FET in the mouse brain was detected by PET to assess tumorigenesis. The expression of ^18^F-FET in mouse brain injected with ATP11B-overexpressing U251 cells was significantly reduced in comparison with the vector group (Fig. 6a, b). ATP11B overexpression inhibited the growth of glioma cells, which is consistent with in vitro observations. To evaluate the role of LINC00606 and ATP11B in the occurrence and development of glioma, a xenograft nude mouse model was used. Firstly, the lentiviral vectors were transfected together with packing plasmids to U251 cells and then subcutaneously injected into nude mice. OE ATP11B and sh-LINC00606 significantly reduced the weight and volume of xenograft tumors in mice (Fig. 6c–h). The effects of LINC00606 and ATP11B on the p-PI3K/PI3K, p-AKT/AKT, p-GSK3β/GSK3β, and PTEN protein levels were determined by Western blotting in vivo*.* Consistent with the results obtained from U251 cells, sh-LINC00606 led to an increase in PTEN expression levels and a suppression of the PI3K/AKT signaling pathway. Overexpression of ATP11B showed a similar regulatory trend (Fig. 6i, Supplementary Fig. S9m-p). Immunohistochemistry (IHC) demonstrates that ATP11B overexpression or LINC00606 knockdown resulted in decreased Ki67 expression in xenograft tumors (Fig. 6j), suggesting suppression of xenografted U251 cell tumorigenicity in vivo. H&E staining of untreated tumor tissues shows the presence of dense cells, severe nuclear atypia, and more prominent angiogenesis and necrosis (Fig. 6k). Therefore, understanding the underlying mechanisms of LINC00606 and/or ATP11B in glioma has novel implications in future therapies to inhibit glioma progression and recurrence.

## Discussion

Glioblastoma, as a malignant tumor, has a poor prognosis despite possessing a favorable methylation profile. This is due to the strong proliferation of glioma cells and high infiltration in the brain, which leads to the failure of localized treatment [43]. Therefore, controlling the proliferation of glioma cells and reducing their invasiveness has become an important research direction. Imbalance in lncRNA expression is closely related to the occurrence and development of cancer [39, 44]. Here, we reveal for the first time that the expression level of LINC00606 is significantly and specifically upregulated in GBM, and that GBM with high LINC00606 expression has more malignant potential and a poorer prognosis. Functionally, LINC00606 can promote the proliferation and migration of glioma cells and reduce the rate of apoptosis. With respect to the molecular mechanism, we found that LINC00606 mainly exists in the cytoplasm and promotes glioma progression by sponging miR-486-3p, which targets TCF12. In addition, the target gene *TCF12* is highly expressed in glioma and acts as a transcription factor to control the transcription of LINC00606, PTEN, and KLLN. Moreover, LINC00606 binds to ATP11B and participates in the regulation of the PI3K/AKT signaling pathway, which decreases the level of apoptosis and thus promotes the progression of glioma.

LncRNAs not only regulate gene expression and participate in signal transduction pathways but also regulate epigenetic, transcriptional, and post-transcriptional modifications [45]. LncRNA expression is closely related to a variety of biological functions, including cell survival, cancer progression and metastasis [46, 47]. Recent studies have shown that abnormal expression levels of lncRNAs are closely related to the pathology and prognosis of glioma [48]. LncRNA LPP-AS2 promoted glioma tumorigenesis via a miR-7-5p/EGFR/PI3K/AKT/c-MYC feedback loop [49]. The lncRNA KB-1460A1.5 regulates TSC1 expression by sponging miR-130a-3p and participates in miR-130a-3p/TSC1/mTOR/YY1 feedback loop to inhibit glioma tumorigenesis [50]. Here, we show that LINC00606 expression is enriched in glioma, including GBM and LGG, and is associated with adverse prognosis, suggesting that LINC00606 can be used as a specific biomarker of glioma. In addition, knockdown of LINC00606 reduces the proliferation and migration of glioma cells and promotes apoptosis. Similarly, our in vivo results also show that decreased expression of LINC00606 inhibits tumor growth, indicating that LINC00606 may become a novel specific target for the treatment of glioma.

We demonstrate the binding sites between LINC00606 and miR-486-3p and confirm a targeted binding relationship. According to Next Generation Sequencing (NGS), miR-486-3p is considered a biomarker to distinguish glioblastoma from low-grade astrocytoma [51] and miR-486-3p may be a potential target for the treatment of glioblastoma to enhance drug sensitivity [52]. MiR-486-3p inhibits the proliferation, migration, and invasion of retinoblastoma, inhibits the proliferation and metastasis of cervical cancer, targets FGFR4 and EGFR to mediate drug resistance in hepatocellular carcinoma, and reverses the malignant phenotype of GBM cells [53–55]. Moreover, miR-486-5p has been shown to regulate GBM stem-like cells through PTEN-dependent signaling pathways [56]; however, the molecular mechanism of miR-486-3p in glioma has not yet been fully revealed. In the present study, we found that miR-486-3p is downregulated in glioblastoma, and its target genes are mainly enriched in transcriptional regulation and the PI3K/AKT signaling pathway. As the target gene of miR-486-3p, the transcription factor TCF12 is significantly differentially expressed in various types of glioma and is associated with a poor prognosis. It is surprising that TCF12 is directly enriched in the promoter region of LINC00606. In addition, TCF12 is also enriched in the promoters of the tumor suppressor PTEN and the apoptotic factor KLLN, affecting them bi-directionally. This positive feedback loop mechanism expands our understanding of the epigenetic modification of LINC00606 in glioma. In addition, we need to further explore the mechanism of transcription factor regulation. Cell fate is determined by co-regulation of transcription factors and epigenetic factors [57]. Whether TCF12 can form complexes on chromatin by recruiting other transcription factors to regulate histone methylation levels of gene promoters and enhancers, thereby regulating transcription activity of downstream genes such as PTEN or KLLN, which in turn affects the occurrence of GBM. This will reveal in more detail that LINC00606, as a key node in the gene regulatory center network in gliomas, participates in the transcription factor-epigenetic regulator pathway and regulates tumor occurrence and development.

The strong proliferation and migration characteristics of glioma cells are the main reason for the low cure rate, postoperative recurrence, and poor prognosis [3]. ATP11B has been shown to be closely associated with tumor metastasis; however, its role in glioma has not yet been elucidated. It demonstrates that the expression of ATP11B in the human malignant glioma cell lines, U251, U87, and U118, is significantly decreased at both the mRNA and protein levels in comparison with that in the normal human glial cell line HEB. ATP11B can affect the proliferation, migration, and colony formation of glioma cells. Additionally, we show that LINC00606 binds directly to ATP11B; more specifically, 1–1584nt of LINC00606 binds to the I1- I4 segments of ATP11B in the cytoplasm to modulate its mRNA and protein expression levels. In addition, using bioinformatics, we uncover that following overexpression of ATP11B in U251 cells, the DEGs are mainly enriched in the PI3K/AKT signaling pathway, which is the same as that for the target gene of miR-486-3p. The PI3K/AKT pathway is essential for the control of cell growth, and its aberrant activation is often responsible for tumorigenesis in many cancers, including glioma [58, 59]. Overexpression of ATP11B and silencing of LINC00606 significantly inhibits the PI3K/AKT pathway and promotes apoptosis of U251 cells. These data provide new insights into the molecular mechanisms of LINC00606 and ATP11B in glioma. Furthermore, the expression of ^18^F-FET in the brains of mice injected with ATP11B-overexpressing U251 cells was significantly reduced as compared with the vector, and OE ATP11B and sh-LINC00606 inhibited the growth of glioma cells in vivo model. Our data provide insights into the regulatory mechanism of LINC00606 in glioma.

In conclusion, our findings provide important clues for further study of the molecular mechanism of LINC00606 and ATP11B in glioma. In addition, the results of this study have important clinical significance. Our study found that LINC00606 is highly expressed in GBM, and inhibition of its expression can hinder the GBM process by experimental verification in vivo and in vitro. This provides a strong basis for translating LINC00606 into clinical practice. Therefore, if the biologics of LINC00606 can be applied to clinical trials, combination therapy with targeted drugs may improve the treatment outcome of GBM patients. We will further explore this possibility in future studies.

## Conclusion

In this study, we identified LINC00606 as a prognostic risk factor in GBM, which is highly expressed in glioma patients and is associated with a malignant prognosis. Mechanistically, LINC00606 functions as a sponge for miR-486-3p to participate in the regulation of tumor malignant progression. Interestingly, the target gene TCF12 of miR-486-3p is involved in the transcriptional initiation process of LINC00606, PTEN, and KLLN. Furthermore, LINC00606 can bind to protein ATP11B, which exerts anti-tumor effects in GBM, and affects the apoptosis level of GBM through the PI3K/AKT signaling pathway axis.

### Supplementary Information

**Supplementary Material 1. **

**Supplementary Material 2. **

## Data Availability

For the miR-486-3p and target genes study, the non-coding RNA profiling data are available in the GEO database at NCBI (https://www.ncbi.nlm.nih.gov/geo/) under accession number GSE113486. The target genes data are available in the RNA22, miR-WALK, miRDB, miRmap, and TargetScan7 databases. URL: https://cm.jefferson.edu/rna22/Interactive; http://mirwalk.umm.uni-heidelberg.de/; http://mirdb.org; https://mirmap.ezlab.org/docs/; https://www.targetscan.org/vert_80/ For the TCF12 study, the gene expression profile data are available in the TCGA/GTEx data are available within GEPIA2 (http://gepia2.cancer-pku.cn/#index). See the results in the text for details. For the RNA binding domains study, the interaction map, protein residue index, RNA-binding domains data are available in catRAPID (http://service.tartaglialab.com/page/catrapid_group).

## Abbreviations

GBM: Glioblastoma
LGG: Low-grade glioma
HEB: Human brain glial cell line
RIP: RNA immunoprecipitation
GO: Gene ontology
KEGG: Kyoto encyclopedia of genes and genomes
DEGs: Differentially expressed genes
TF: Transcription factors
PRMT5: Protein arginine methyltransferase 5
NGS: Next Generation Sequencing
AGO2: Argonaute2 protein
^18^F-FET: 18F-fluoroethyl-tyrosine
CHIRP: Chromatin Isolation by RNA Purification
WT: Wild-type
Mut: Mutation
H&E: Hematoxylin and eosin
