# Supplementary material for: LINC00606 promotes glioblastoma progression through sponge miR-486-3p and interaction with ATP11B
Source: J Exp Clin Cancer Res. 2024 May 9;43:139. doi: 10.1186/s13046-024-03058-z (PMC11080186; doi:10.1186/s13046-024-03058-z)
Supplement: Supplementary file 1 — Supplementary Material 1. [file 13046_2024_3058_MOESM1_ESM.docx]

**Supplementary Material for**

LINC00606 promotes glioblastoma progression through sponge miR-486-3p and interaction with ATP11B

Naijun Dong^1,2 #^, Wenxin Qi^1,2 #^, Lingling Wu^1,2^, Jie Li^3^, Xueqi Zhang^1^, Hao Wu^1^, Wen Zhang^1^, Jiawen Jiang^1^, Shibo Zhang^1^, Wenjun Fu^1^, Qian Liu^1^, Guandong Qi^7^, Lukai Wang^7^, Yanyuan Lu^7^, Jingyi Luo^7^, Yanyan Kong^9^, Yihao Liu^8*^ Robert Chunhua Zhao^1,4,5,6 *^, and Jiao Wang^1 *^

Correspondence to Yihao Liu **(**[xb88053@sjtu.edu.cn](mailto:xb88053@sjtu.edu.cn)**)**, Robert Chunhua Zhao ( [zhaochunhua@vip.163.com](mailto:zhaochunhua@vip.163.com)) & Jiao Wang ([jo717@shu.edu.cn](mailto:jo717@shu.edu.cn))

**This PDF file includes:**

Supplementary Figure S1-9 with their legends

Supplementary Table S1-7


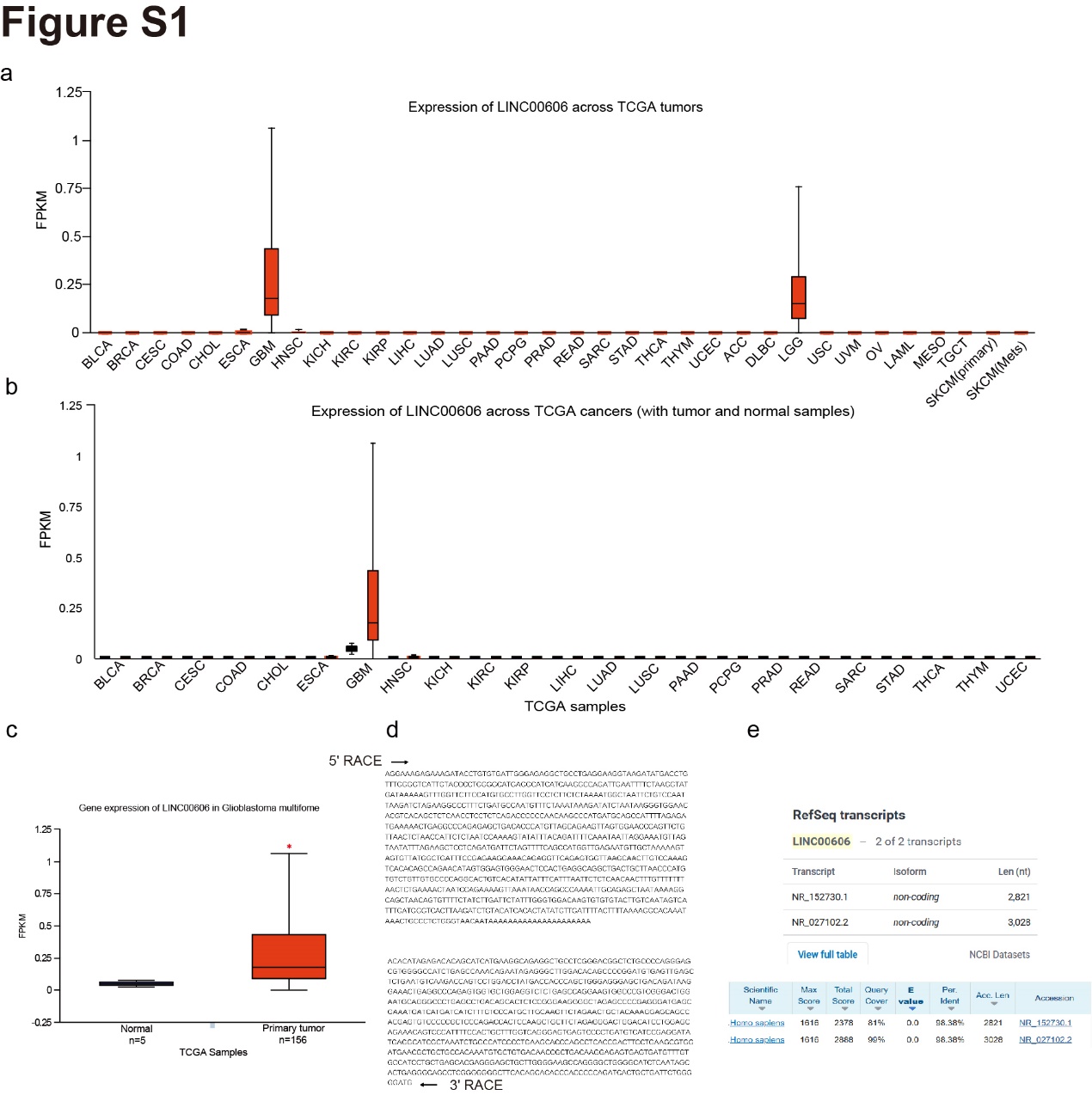


**Supplementary Fig. S1. Expression pattern of LINC00606 according to the pan-cancer and GBM databases.**

(**a**) The pan-cancer expression level of LINC00606 was analyzed in the TCGA database [1].

(**b, c**) Analysis of glioma and normal brain tissues in the TCGA database shows that the expression of LINC00606 was significantly higher in glioma than normal brain tissues (normal: n = 5; primary tumors: n = 156) [1].

(**d**) Left: The 5′-3′ RACE PCR sequencing results for LINC00606. Top: sequencing results for 5′ RACE; bottom: sequencing results for 3′ RACE (only some of the sequencing results are displayed). Right: Information about the LINC00606 transcripts.

(**e**) Information about the LINC00606 transcripts.

Data are expressed as the mean ± SD of three independent experiments. **P* < 0.05; ***P* < 0.01; ****P* < 0.001.


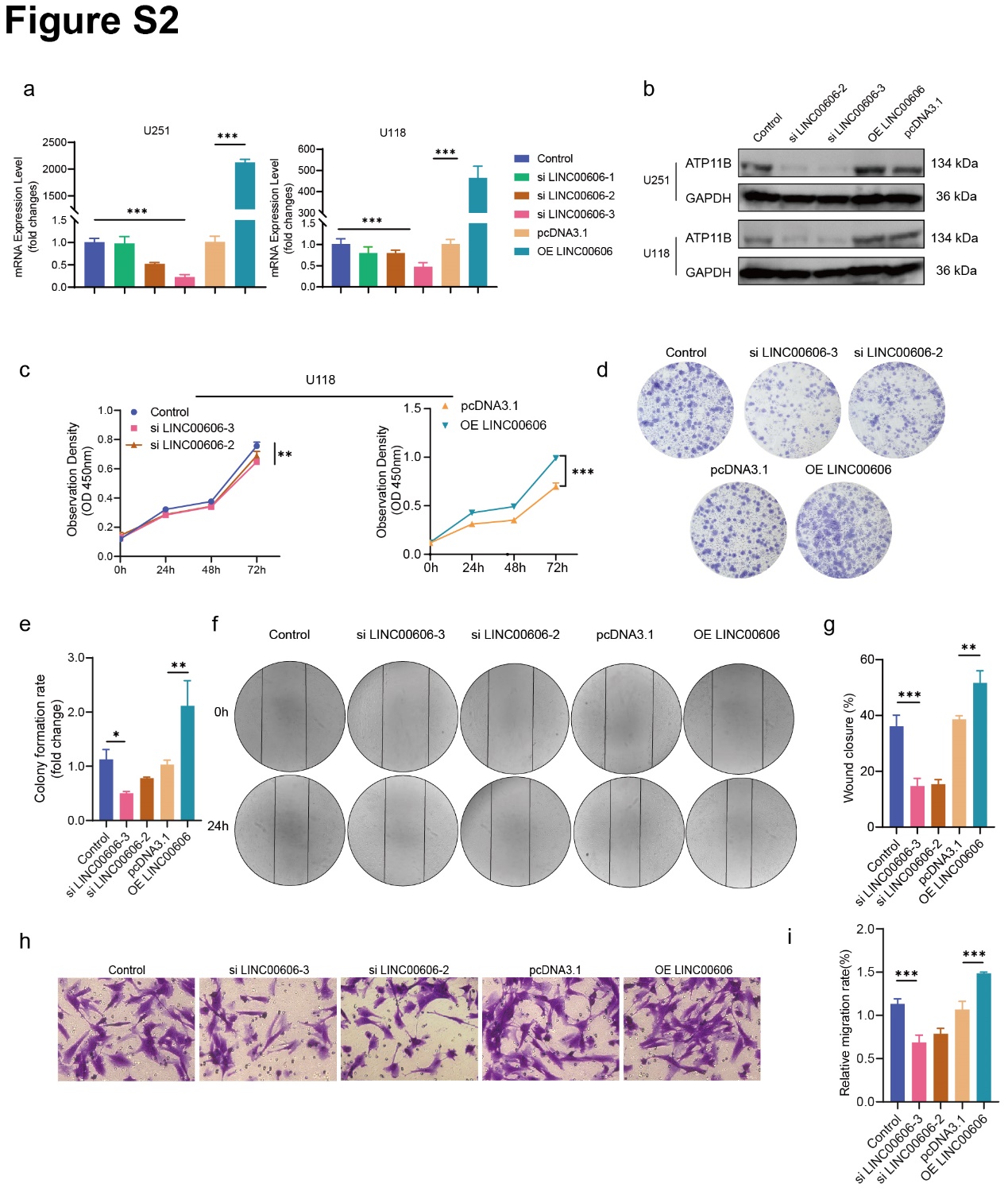


**Supplementary Fig. S2. Knockdown of LINC00606 hinders the tumor-promoting functions of U118 cells.**
(**a, b**) The expression level of LINC00606 following transfection of U251 and U118 cells with control siRNA (Control), LINC00606 siRNA (siLINC00606), control plasmid (pcDNA3.1), and LINC00606-overexpression plasmid (OE LINC00606).

(**c**) CCK-8 assay was performed to evaluate the proliferation rate of U118 cells following transfection with control siRNA (Control), LINC00606 siRNA (siLINC00606), control plasmid (pcDNA3.1), and LINC00606-overexpression plasmid (OE LINC00606). The OD value was measured at 450 nm.
(**d, e**) Colony formation assay showing that LINC00606 overexpression in U118 cells promoted cell proliferation. Statistical analysis of colony numbers was performed using Image J.

(**f, g**) Wound-healing assay was performed to detect the migration ability of U118 cells following transfection with si LINC00606 or OE LINC00606. Photos were taken at 0 and 24 h. A histogram was used for statistical analysis of wound-healing.

(**h, i**) Transwell assay was performed to detect the migration ability of U118 cells following transfection with si LINC00606 or OE LINC00606. The number of migrated cells was analyzed using Image J. Scale bars represent 50 μm.

Data are expressed as the mean ± SD of three independent experiments. **P* < 0.05; ***P* < 0.01; ****P* < 0.001.


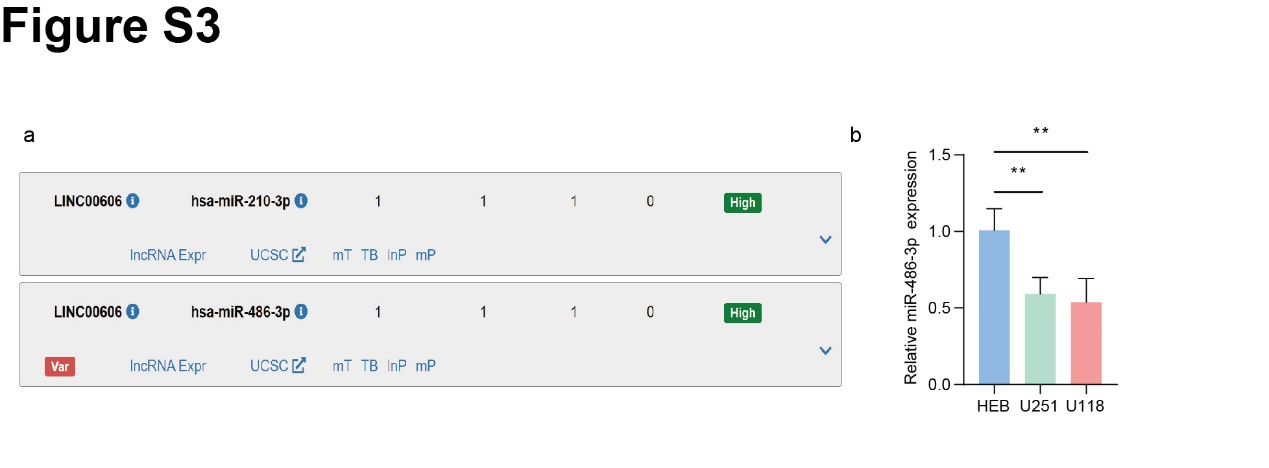


**Supplementary Fig. S3. Target miRNA prediction for LINC00606.**

(**a**) DIANA Tools miRNA–lncRNA results showing two interactions for LINC00606: miR-210-3p and miR-486-3p.

(**b**) QPCR: The expression level of miR-486-3p in HEB, U251 and U118 cells.

Data are expressed as the mean ± SD of three independent experiments. **P* < 0.05; ***P*< 0.01;


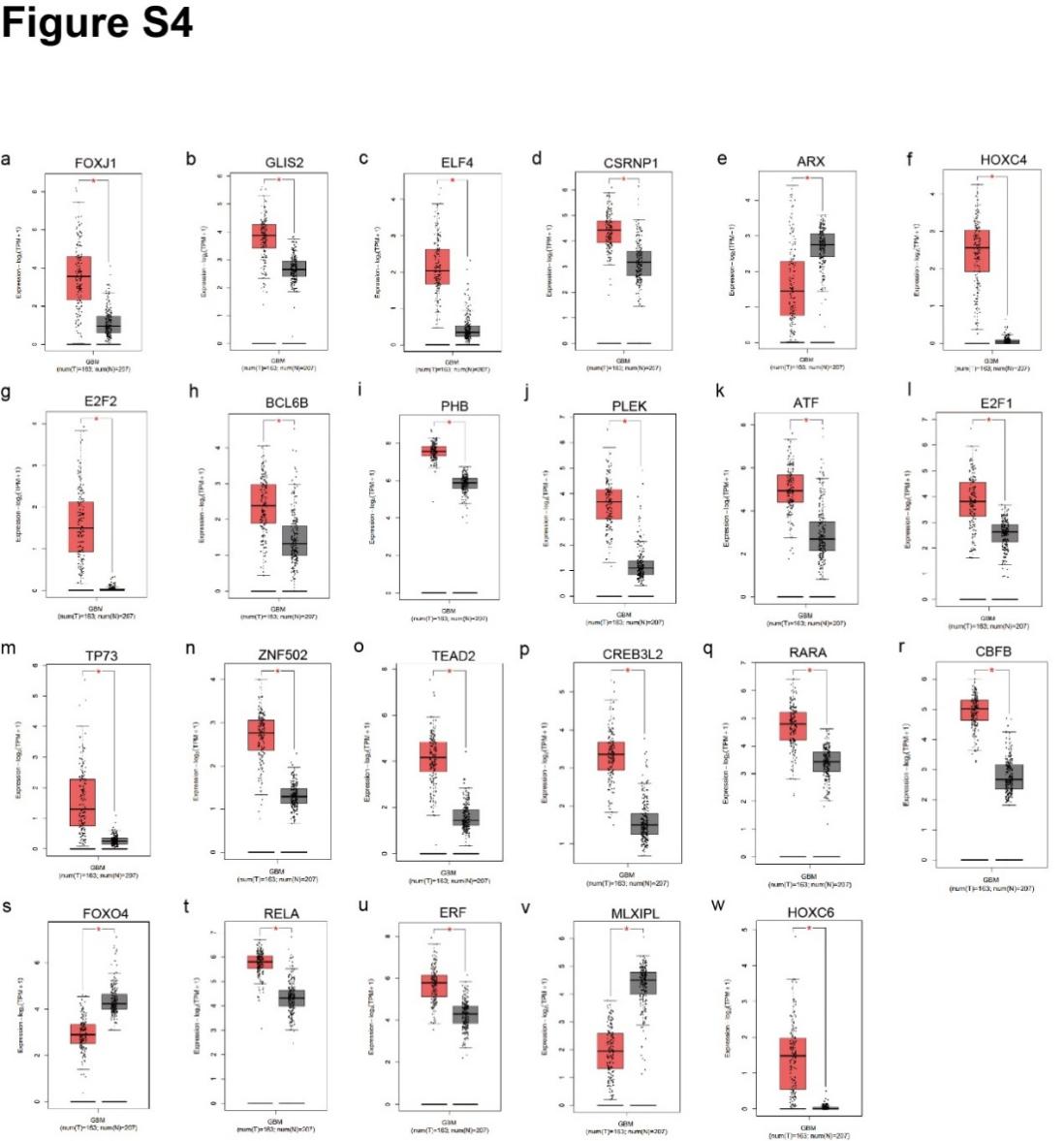


**Supplementary Fig. S4. In the TCGA and GTEx databases, 23 transcription factors are differentially expressed between GBM and normal tissues.**

(**a–w**) The TCGA and GTEx database was used to analyze the differential expression of 23 transcription factors between GBM (n = 163) and normal (n = 207) tissue samples. The 23 transcription factors include: ARX, ATF, BCL6B, CBFB, CREB3L2, CSRNP1, E2F1, E2F2, ELF4, ERF, FOXJ1, FOXO4, CLIS2, HOXC4, HOXC6, MLXIPL, PHB, PLEK, RALA, RARA, TEAD2, TP73, and ZNF502. The results of TCF12 are shown in Fig. 4e.


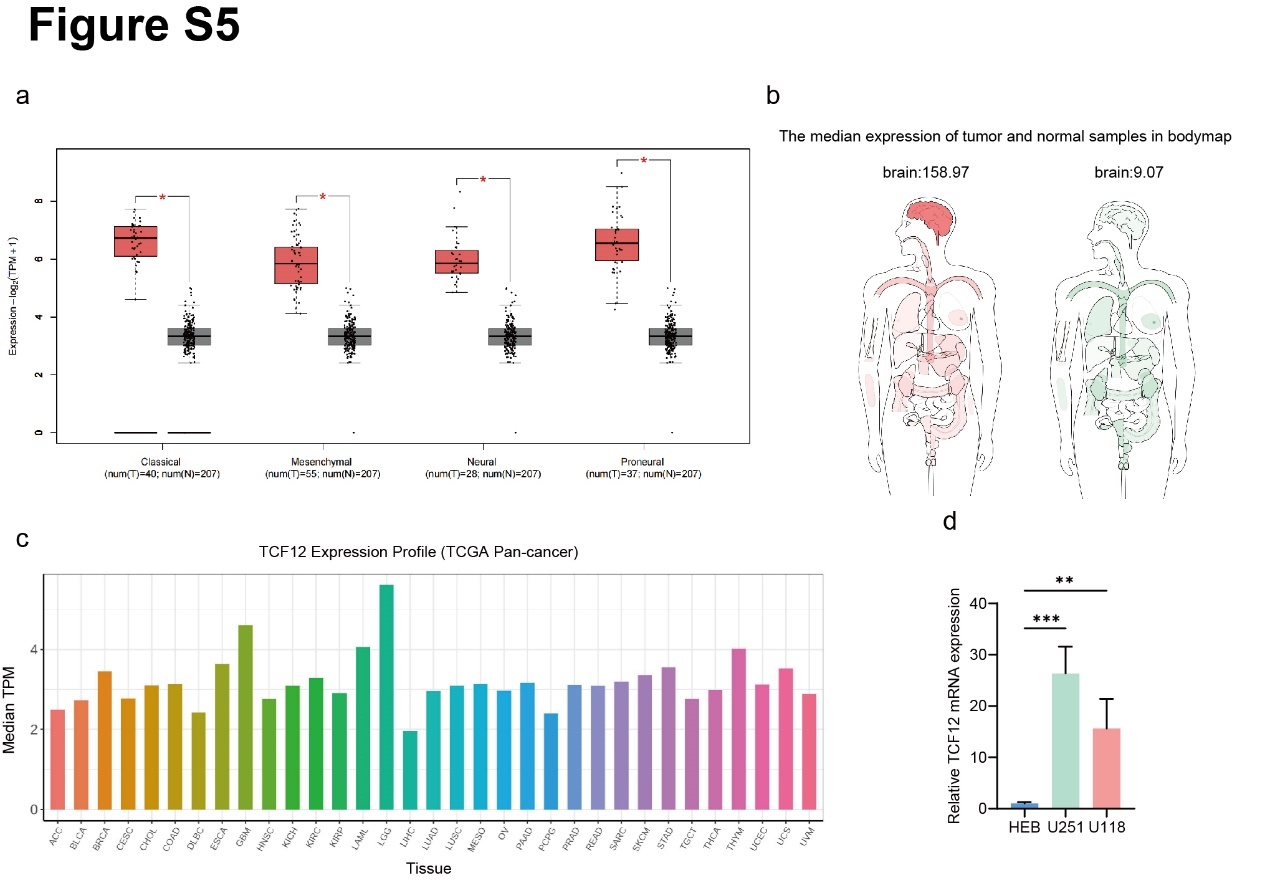


**Supplementary Fig. S5. Analysis of differentially expressed target genes in the TCGA and GTEx databases.**

(**a**) The TCGA and GTEx databases were used to analyze the expression of TCF12 in four different subtypes of GBM.

(**b**) The median expression of tumor and normal samples shown as a body map, according to data from the TCGA and GTEx databases.

(**c**) The pan-cancer expression of TCF12 was analyzed using the TCGA and GTEx databases.

(**d**) QPCR: The expression level of TCF12 in HEB, U251 and U118 cells. Data are expressed as the mean ± SD of three independent experiments. **P* < 0.05; ***P* < 0.01; ****P* < 0.001.


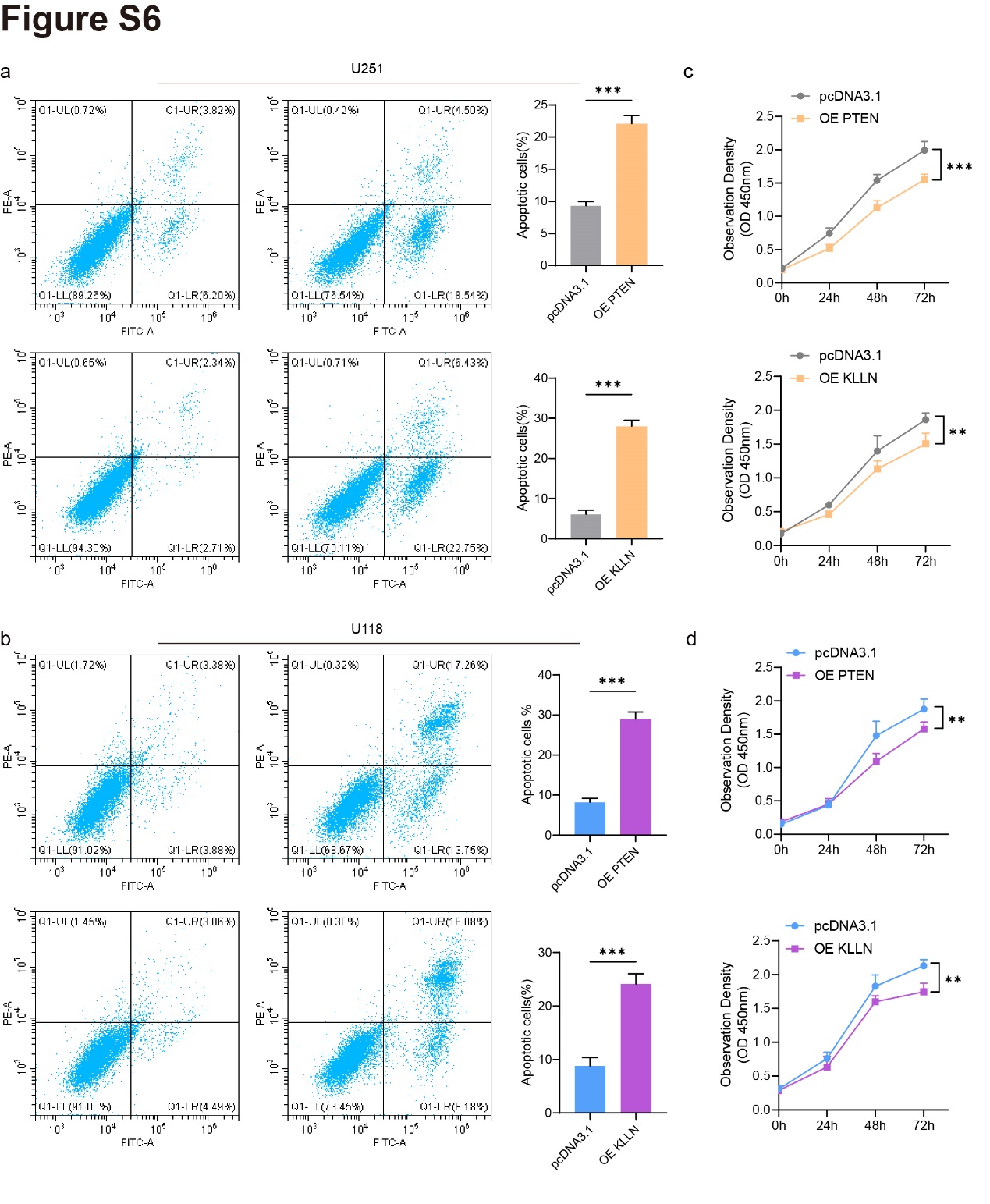


**Supplementary Fig. S6. PTEN and KLLN attenuate the proliferation of glioma cells and promote apoptosis**

(**a b**) Flow cytometry was used to analyze the apoptosis level of U251 and U118 cells with overexpression of PTEN or KLLN.

(**c d**) CCK-8 assay was performed to evaluate the proliferation rate of U251 and U118 following transfection with OE PTEN, KLLN or pcDNA3.1. The OD value was measured at 450 nm.

Data are expressed as the mean ± SD of three independent experiments. **P* < 0.05; ***P* < 0.01; ****P*< 0.001.


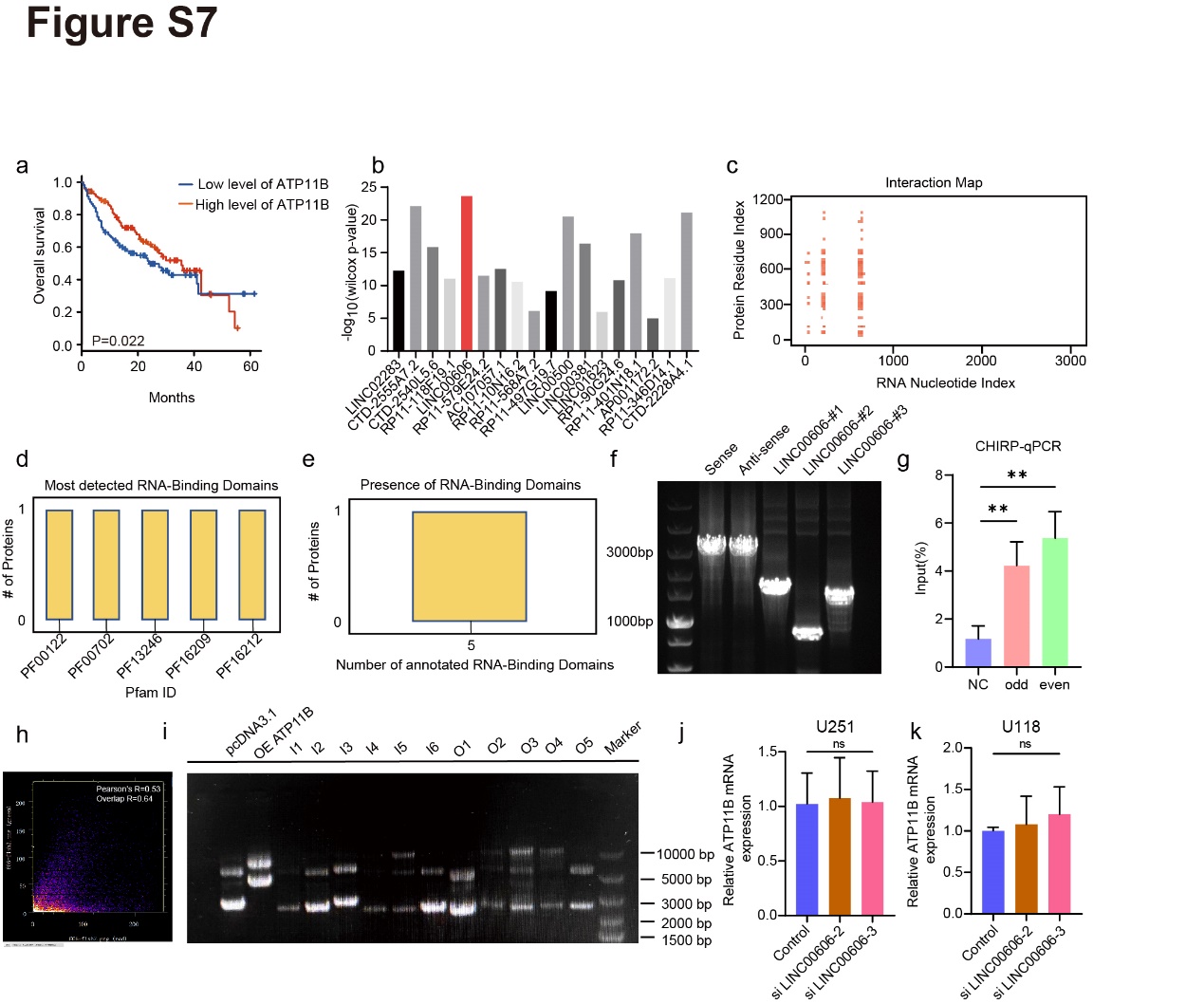


**Supplementary Fig. S7. Relationship between the RNA-binding domains and the protein interaction of LINC00606 and ATP11B.**

(**a**) Kaplan–Meier analysis of the overall survival of 120 GBM patients in the high-level ATP11B (n = 47) and low-level ATP11B (n = 73) groups.

(**b**) Analysis of the relative expression levels of lncRNAs in U251 cells according to RIP-seq (Wilcoxon test).

(**c**) Interaction map of the RNA nucleotide index (LINC00606) and protein residue index (ATP11B) as predicted by catRAPID.

(**d, e**) RNA-binding domains in LINC00606 and ATP11B as predicted by catRAPID.

(**f**) Agarose gel electrophoresis of successfully constructed LINC00606 PCR products truncated according to the secondary structure.

(**g**) Analysis of CHIRP-qPCR results in NC group, odd group and even group.

(**h**) Automatic quantitative analysis of co-localization of FISH-LINC00606/ATP11B.

(**i**) Agarose gel electrophoresis of successfully constructed intracellular and extracellular PCR products of ATP11B truncated according to the transmembrane domains.

(**j, k**) The expression level of ATP11B following transfection of U251 and U118 cells with control siRNA (Control), LINC00606 siRNA-2 or LINC00606 siRNA-3.

Data are expressed as the mean ± SD of three independent experiments. **P* < 0.05; ***P* < 0.01.


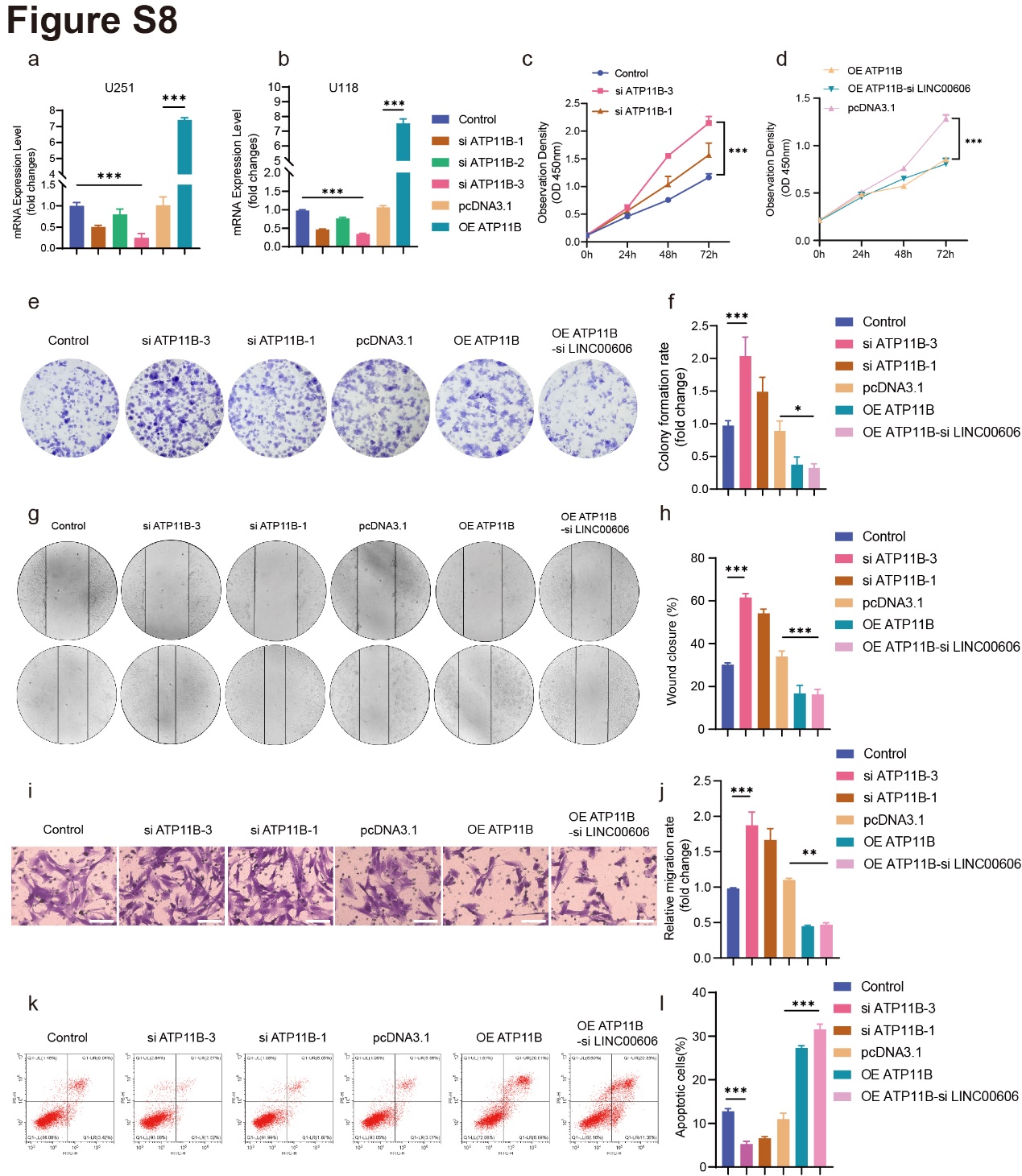


**Supplementary Fig. S8. LINC00606 overexpression promotes the malignant behavior of U118 cells by decreasing ATP11B expression.**

(**a, b**) The expression level of ATP11B following transfection of U251 and U118 cells with control siRNA (Control), ATP11B siRNA (si ATP11B), control plasmid (pcDNA3.1), and ATP11B-overexpression plasmid (OE ATP11B).

(**c**) The CCK-8 assay was applied to evaluate the proliferation rate of U118 cells following transfection with control siRNA (Control) or ATP11B siRNA (si ATP11B).

(**d**) The CCK-8 assay was applied to evaluate the proliferation rate of U118 cells following transfection with the control plasmid (pcDNA3.1) or the ATP11B-overexpression plasmid (OE ATP11B) and co-transfection of the ATP11B-overexpression plasmid and LINC00606 siRNA.

(**e, f**) Colony formation assay of U118 cells showing that knockdown of ATP11B promotes cell proliferation. Statistical analysis of colony number was performed using Image J.

(**g, h**) Wound-healing assay was used to detect the migration ability of U118 cells with or without si ATP11B and OE ATP11B. Photos were taken at 0 and 24 h. A histogram was used for statistical analysis of wound-healing.

(**i, j**) Transwell assay was performed to detect the migration ability of U118 cells with or without siATP11B and OE ATP11B. The number of migrated cells was analyzed using Image J. Scale bars represent 100 μm.

(**k, l**) Flow cytometry was used to analyze the apoptosis level of U118 cells with or without knockdown and overexpression of ATP11B.

Data are expressed as the mean ± SD of three independent experiments. **P* < 0.05; ***P* < 0.01; ****P*< 0.001.


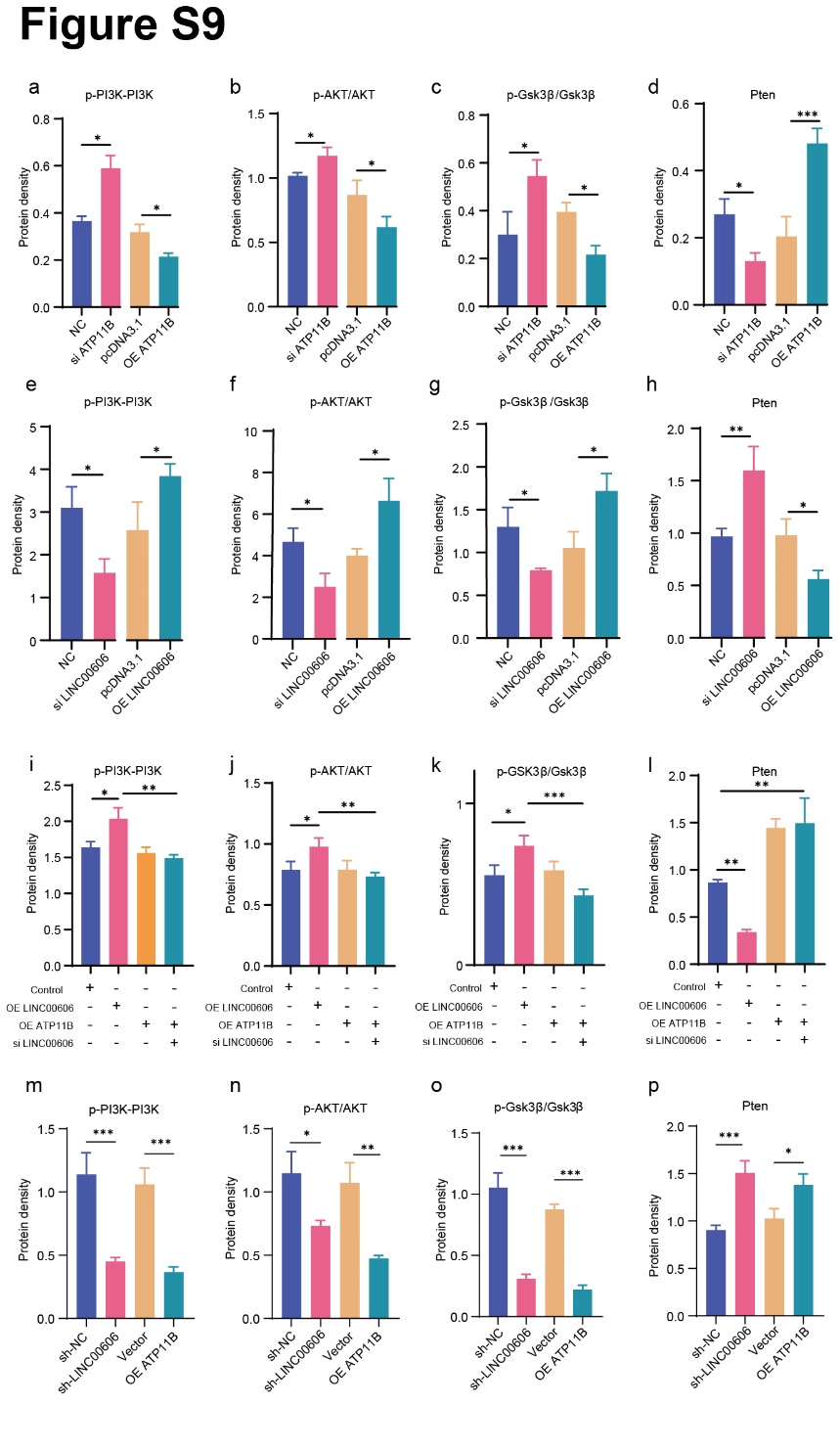


**Supplementary Fig. S9. Quantitative analysis of protein expression.**

(**a–p**) Immunoblotting of p-PI3K, PI3K, p-AKT, AKT, p-GSK3β, GSK3β, and PTEN expression levels regulated by ATP11B and LINC00606 in U251 cells. Endogenous vinculin or GAPDH was used as the internal control.

Data are expressed as the mean ± SD of three independent experiments. **P* < 0.05; ***P* < 0.01; ****P* < 0.001.

**Supplementary Table S1: Summary of clinical GBM patients**

| **Characteristic** | **All patient** | |
| --- | --- | --- |
|  | **Low**  **LINC00606 expression**  **(n=42)** | **High**  **LINC00606 expression**  **(n=78)** |
| **Type** |  |  |
| Pri GBM | 30 | 48 |
| Rec GBM | 12 | 30 |
| **Sex (n)** |  | |
| Male | 27 | 45 |
| Female | 15 | 33 |
| **age** |  |  |
| ≥45 | 29 | 59 |
| ＜45 | 13 | 19 |
| **Tumor location** |  | |
| Frontal | 22 | 40 |
| Non-frontal | 20 | 38 |
| **KPS score** |  |  |
| ≥80 | 34 | 60 |
| ＜80 | 8 | 18 |
| **Extent of surgery** |  |  |
| Total | 33 | 64 |
| Subtotal | 9 | 14 |

**Abbreviations**: KPS, Karnofsky performance status.

**Supplementary Table S1: Summary of clinical GBM patients**

| **Characteristic** | **All patient** | |
| --- | --- | --- |
|  | **Low**  **ATP11B expression**  **(n=73)** | **High**  **ATP11B expression**  **(n=47)** |
| **Type** |  |  |
| Pri GBM | 41 | 26 |
| Rec GBM | 32 | 21 |
| **Sex (n)** |  | |
| Male | 44 | 33 |
| Female | 29 | 14 |
| **age** |  |  |
| ≥45 | 50 | 30 |
| ＜45 | 23 | 17 |
| **Tumor location** |  | |
| Frontal | 46 | 22 |
| Non-frontal | 27 | 25 |
| **KPS score** |  |  |
| ≥80 | 60 | 31 |
| ＜80 | 13 | 16 |
| **Extent of surgery** |  |  |
| Total | 39 | 29 |
| Subtotal | 34 | 18 |

**Abbreviations**: KPS, Karnofsky performance status.

**Supplementary Table S2: The sequences of siRNAs**

| **Name** | **Target sequence** |
| --- | --- |
| **genOFFTM st-h-ATP11B_001** | **GGAGTACTATGCATCTTCA** |
| **genOFFTM st-h-ATP11B_002** | **GGGTGATATTGTTCGAATA** |
| **genOFFTM st-h-ATP11B_003** | **GAAACGATCTGCAGTAGAA** |
| **LINC00606-ASO1** | **A*T*G*A*CAACTTTGTCAT*T*A*G*C** |
| **LINC00606-ASO2** | **C*A*G*A*GCTAGATCAAGA*A*A*T*G** |
| **LINC00606-ASO3** | **A*T*G*A*TCATGATCATTT*C*G*C*T** |
| **LINC00606-siRNA1-F** | **GGAUAUGAGGCAUCGCUAATT** |
| **LINC00606-siRNA1-R** | **UUAGCGAUGCCUCAUAUCCTT** |
| **LINC00606-siRNA2-F** | **GGUUCAGAGUGGUUAAGCATT** |
| **LINC00606-siRNA2-R** | **UGCUUAACCACUCUGAACCTT** |
| **LINC00606-siRNA3-F** | **GUCCAAUUAAGAUCUAGAATT** |
| **LINC00606-siRNA3-R** | **UUCUAGAUCUUAAUUGGACTT** |

**Supplementary Table S3: List of PCR and qPCR primers used in this study**

| **Primers for CHIP-qPCR (Name ID)** |  |
| --- | --- |
| **LINC00606-0kb-F** | **CTAGTCCCTAGGCCACCTGT** |
| **LINC00606-0kb-R** | **CTCTGGGGTTGGAGCAGAAG** |
| **LINC00606-(+1kb)-F** | **GAGTCAGCGAGAGCTGTGTT** |
| **LINC00606-(+1kb)-R** | **AGAAACAGGTGGCCTAGGGA** |
| **LINC00606-(+4kb)-F** | **TCCAGCTGACTTCGGTGAAC** |
| **LINC00606-(+4kb)-R** | **CTGGAGGGGAAATGGCTCAG** |
| **LINC00606-(-3kb)-F** | **TGCAAGAACGACCTTGGGAC** |
| **LINC00606-(-3kb)-R** | **GAGCATGAGGAAACTCGGCA** |
| **LINC00606-(-5kb)-F** | **TGCCGTGCAAGTGACACATA** |
| **LINC00606-(-5kb)-R** | **ATTGGGACATGGCAAGTGCT** |
| **PTEN-KLLN-0kb-F** | **CACGTGACCTCCTTCGGAAA** |
| **PTEN-KLLN-0kb-R** | **TCTCAGAGACCACCTAGCCC** |
| **PTEN-KLLN-(+1kb)-F** | **TTTTAGGGCAAACGAGCCGA** |
| **PTEN-KLLN-(+1kb)-R** | **GCCAGCGTGTATCACCTCAT** |
| **PTEN-KLLN-(+3kb)-F** | **ATGCCAGAATTGCTGCTCAC** |
| **PTEN-KLLN-(+3kb)-R** | **AGTAAACCCCTCTTGCCAGC** |
| **PTEN-KLLN-(-3kb)-F** | **AAGTGCTTGGAGACCAGTGT** |
| **PTEN-KLLN-(-3kb)-R** | **CCAAATGGCTGGCCCTATCT** |
| **PTEN-KLLN-(-5kb)-F** | **GTGGGCTGGGAAGAAGTTGT** |
| **PTEN-KLLN-(-5kb)-R** | **CATCCTCTTGAGGGTAGGGGA** |

**Supplementary Table S3: List of PCR and qPCR primers used in this study**

| **Primers for qPCR (Name ID)** |  |
| --- | --- |
| **RNU6-F** | **TGGAACGATACAGAGAAGATTAGCA** |
| **RNU6-R** | **TATGGAACGCTTCACGAATTTGC** |
| **GAPDH-F** | **GTATCGTGGAAGGACTCATGAC** |
| **GAPDH-R** | **ACCACCTTCTTGATGTCATCAT** |
| **ATP11B-F** | **GAAGTGGTGGCCTTGTAAAAACT** |
| **ATP11B-R** | **CCATCCAGTCGATCTGAGGAC** |
| **LINC00606-F** | **AAATGTGCTGTGACAACCGC** |
| **LINC00606-R** | **TGCTATTGAGATGCCCCAGC** |
| **TCF12-F** | **CCAGTAGTTATGGCAACCTTCAT** |
| **TCF12-R** | **GACTCGTGTTTATGTCTGTTGGT** |
| **PTEN-F** | **TTTGAAGACCATAACCCACCAC** |
| **PTEN-R** | **ATTACACCAGTTCGTCCCTTTC** |
| **miR-210-3p-F** | **GCCCCTGCCCACCGCACACT** |
| **miR-210-3p-R** | **ATCAGCCGCTGTCACACGCA** |
| **miR-486-3p-F** | **CGCGGGGCAGCTCAGTA** |
| **miR-486-3p-R** | **AGTGCAGGGTCCGAGGTATT** |
| **pri-miR-486-3p-F** | **GCCGGGGCAGCCAGAC** |
| **pri-miR-486-3p-R** | **AGTGCAGGGTCCGAGGTATT** |
| **pre-miR-486-3p-F** | **CCTCGGGGCAGCTCAGTAC** |
| **pre-miR-486-3p-R** | **AGTGCAGGGTCCGAGGTATT** |

**Supplementary Table S3: List of PCR and qPCR primers used in this study**

| **Primers for qPCR (Name ID)** |  |
| --- | --- |
| **LINC00606-#1-F** | **TAATACGACTCACTATAGGGAGAACACATAGAGACACAGCATCATG** |
| **LINC00606-#1-R** | **TGGGGCAAAATGACTTCATT** |
| **LINC00606-#2-F** | **TAATACGACTCACTATAGGGAGAGGTGCTGGAGGTCTCTGAG** |
| **LINC00606-#2-R** | **GATGGGGGCTGGTGAGCC** |
| **LINC00606-#3-F** | **TAATACGACTCACTATAGGGAGAGGTCACCCAACCAGCGAG** |
| **LINC00606-#3-R** | **ATTGTTACCCAGAGGGCAGT** |
| **LINC606-sense-F** | **TAATACGACTCACTATAGGGAGAACACATAGAGACACAGCATCATG** |
| **LINC606-sense-R** | **ATTGTTACCCAGAGGGCAGTTTATT** |
| **LINC606-Antisense-F** | **ACACATAGAGACACAGCATCATG** |
| **LINC606-Antisense-R** | **TAATACGACTCACTATAGGGAGAATTGTTACCCAGAGGGCAGTTTATT** |

**Supplementary Table S4: Details of sequences used in CHIRP and FISH**

| **Name** | **Sequences** |
| --- | --- |
| **ChIRP-LINC00606**  **Probe-1（odd）** | **TGATCATTTCGCTCATCCCT-/3bio/** |
| **ChIRP-LINC00606**  **Probe-2（even）** | **GATGTCCAGTCCCTCTAGAA-/3bio/** |
| **ChIRP-LINC00606**  **Probe（NC）** | **TCATCGTATTTCGACCCTCT-/3bio/** |
| **LINC00606-FISH** | **AACAGAAGAGAGAGACACTCAGTAGGGG** |
| **signal probe-FISH** | **tt ATGATGATGT ATGATGATGT** |

**Supplementary Table S5: List of Antibodies used in this study**

| **Antibody** | **Company** | **Cat. No.** | **Species** |
| --- | --- | --- | --- |
| **p-PI3K** | **Cell signaling** | **4228T** | **Rabbit** |
| **p-PI3K** | **Abways** | **CY6428** | **Rabbit** |
| **PI3K** | **Proteintech** | **67644-1-Ig** | **Mouse** |
| **p-AKT** | **Abways** | **CY6569** | **Rabbit** |
| **AKT** | **Abways** | **CY5561** | **Rabbit** |
| **p-GSK3β** | **Wanleibio** | **WL03518** | **Rabbit** |
| **GSK3β** | **Cell signaling** | **12456T** | **Rabbit** |
| **GSK3β** | **ABclonal** | **A2081** | **Rabbit** |
| **PTEN** | **ABclonal** | **A19104** | **Rabbit** |
| **TCF12** | **Cell signaling** | **11825S** | **Rabbit** |
| **ATP11B** | **Invitrogen** | **PA5-20996** | **Rabbit** |
| **ATP11B** | **Signalway antibody** | **27644** | **Rabbit** |
| **ATP11B** | **NOVUS** | **NBP1-77190** | **Rabbit** |
| **Ki67** | **Wanleibio** | **WL01384a** | **Rabbit** |
| **Argonaute-2** | **ABclonal** | **A19709** | **Rabbit** |
| **IgG** | **ABclonal** | **AC011** | **Mouse** |
| **GAPDH** | **Abways** | **AB0036** | **Rabbit** |
| **Vinculin** | **Abways** | **CY5164** | **Rabbit** |

**Supplementary Table S6: Details of inserted sequences used in Dual luciferase activity assay**

| **Name** | **Sequences** |
| --- | --- |
| **LINC00606-WT** | **ggtaccTGCAGTTGGTGCCAAATGTCTCCCACCTACCTGCCTGGGTCTCTGCCCCTCAGCTTCTCACAAGTGGCTGTGTTCTTCCACTCACCTGCTGGGACACacacctcgag** |
| **LINC00606-MUT** | **ggtaccTGCAGTTGGTGCCAAATGTCTCCCACCTACCTGaagtttgagagtaaaaTCAGCTTCTCACAAGTGGCTGTGTTCTTCCACTCACCTGCTGGGACACacacctcgag** |
| **TCF-WT** | **ggtaccGAACATAACAAAACCCAGTAGTCACCAAGGCAGGTAGTGTGATAAATGAACACACCACTCTGAGGCTAATTACCTAATGGAATACAAGAGCAATGGTCACCCGTATTTCCTTATCCTAGCCTTTATTTCTCTGTCATTTGGATGGCTGGTCAATGGGGAAGAATTGAGTGGGTGATTTAATCAACTGCAAACCATCTGCCCCTGTCCCAAAATGATGAGCCAGATTAGCATTAAACCAGTACTTGTCAGTCCATCTTAATACTGTTCATTAAGGCACTCTCTGTCTCTAATCCTTAGGAGTTGTTTTAAAAGACATAATCACTTTGAACTTCCATGAAACCTGTCTTCCACCACAACAACctcgag** |
| **TCF-MUT** | **ggtaccGAACATAACAAAACCCAGTAGTCACCAAGGCAGGTAGTGTGATAAATGAACACACCACTCTGAGGCTAATTACCTAATGGAATACAAGAGCAATGGTCACCCGTATTTCCTTATCCTAGCCTTTATTTCTCTGTCATTTGGATGGCTGGTCAATGGGGAAGAATTGAGTGGGTGATTTAATCAACTGCAAACCATAGTAAAATGTCCCAAAATGATGAGCCAGATTAGCATTAAACCAGTACTTGTCAGTCCATCTTAATACTGTTCATTAAGGCACTCTCTGTCTCTAATCCTTAGGAGTTGTTTTAAAAGACATAATCACTTTGAACTTCCATGAAACCTGTCTTCCACCACAACAACctcgag** |

**Supplementary Table S7 Details of candidate proteins**

| **PG. Gene** | **PG. Coverage** | **Unique Peptides** |
| --- | --- | --- |
| **SKIC2**  **ATP11B**  **RFC1**  **DDX42**  **DDX24**  **CHAMP1**  **WASHC5**  **VCPIP1**  **SUGP2**  **CEBPZ**  **NOMO2**  **KRT6C**  **MYO1E**  **STK10**  **EIF3C**  **TAOK1**  **SUPT5H**  **RBM6** | **25.70%**  **29.10%**  **27.30%**  **38.50%**  **36.20%**  **36.80%**  **30%**  **30.60%**  **29.90%**  **33.50%**  **33.40%**  **55.50%**  **27.80%**  **30.10%**  **37.30%**  **30.50%**  **40.80%**  **33.40%** | **26**  **26**  **26**  **27**  **28**  **28**  **28**  **29**  **29**  **31**  **31**  **31**  **31**  **31**  **32**  **34**  **36**  **37** |

**References**

1. Zhang Y, Chen F, Chandrashekar DS, Varambally S, Creighton CJ. Proteogenomic characterization of 2002 human cancers reveals pan-cancer molecular subtypes and associated pathways. Nat Commun. 2022;13(1):2669.
