# Supplementary material for: LINC00606 promotes glioblastoma progression through sponge miR-486-3p and interaction with ATP11B
Source: J Exp Clin Cancer Res. 2024 May 9;43:139. doi: 10.1186/s13046-024-03058-z (PMC11080186; doi:10.1186/s13046-024-03058-z)

Fig 3 h

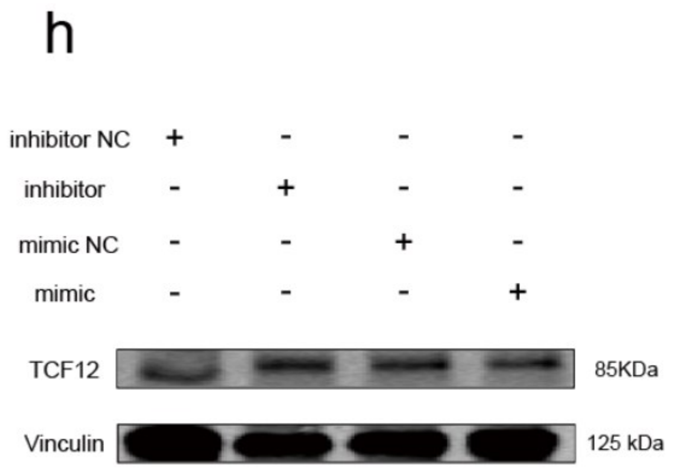
h
inhibitor NC + - - -
inhibitor - + - -
mimic NC - - + -
mimic - - - +
TCF12
85KDa
Vinculin
125 kDa

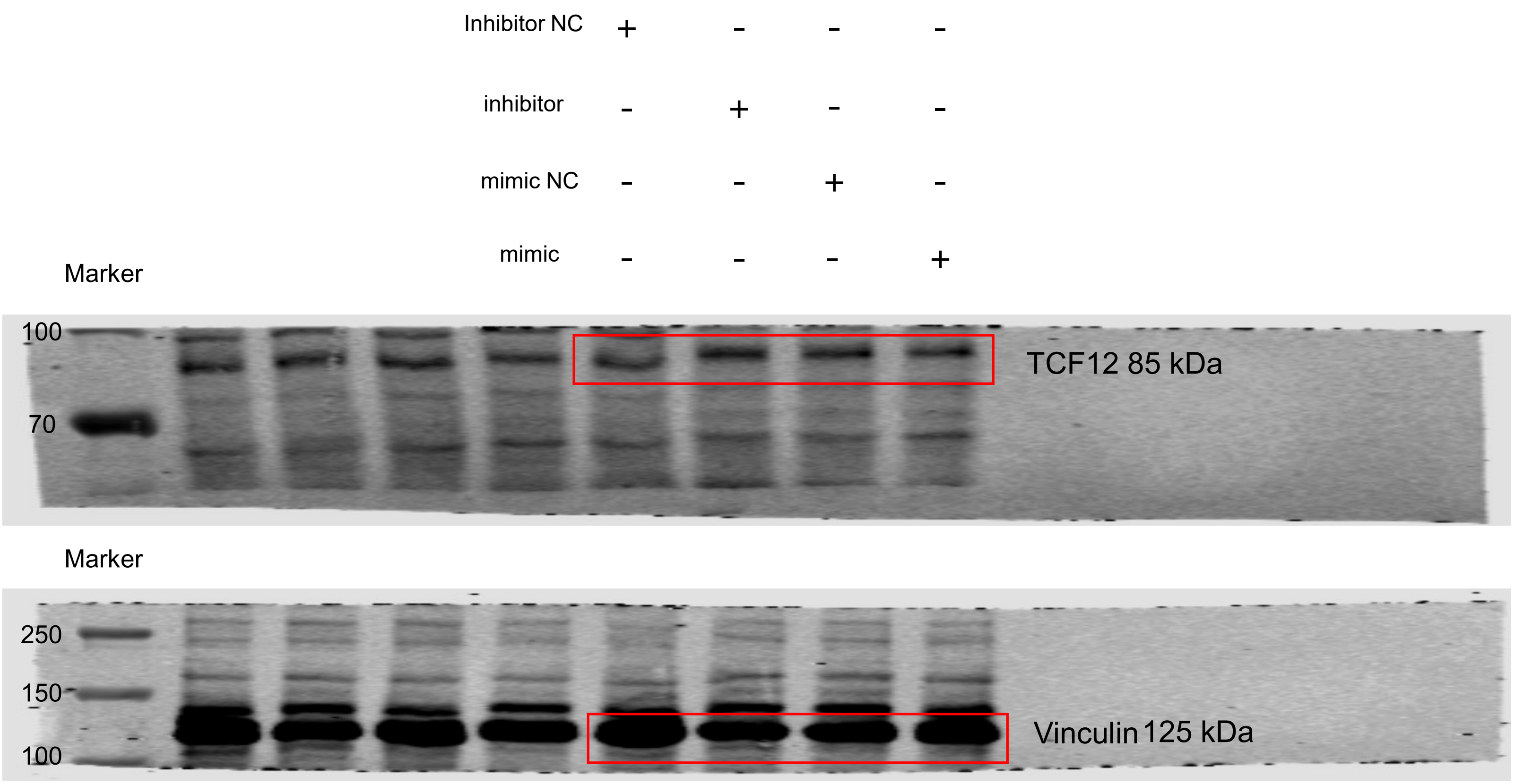
Inhibitor NC + - - -
inhibitor - + - -
mimic NC - - + -
mimic - - - +
Marker
100
70
TCF12 85 kDa
Marker
250
150
100
Vinculin 125 kDa

Fig 3 n

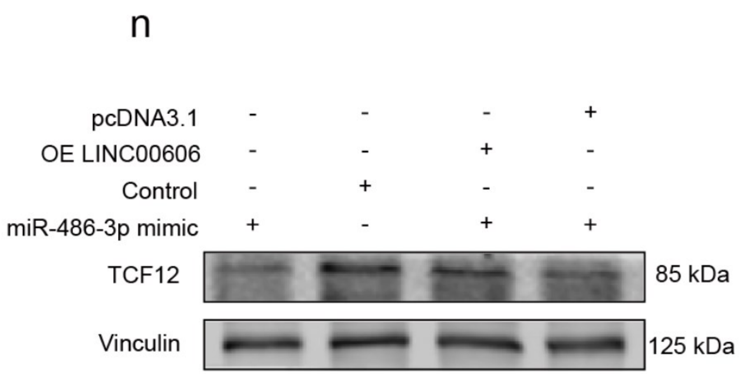

n
pcDNA3.1 - - - +
OE LINC00606 - - + -
Control - + - -
miR-486-3p mimic + - + +
TCF12
85 kDa
Vinculin
125 kDa

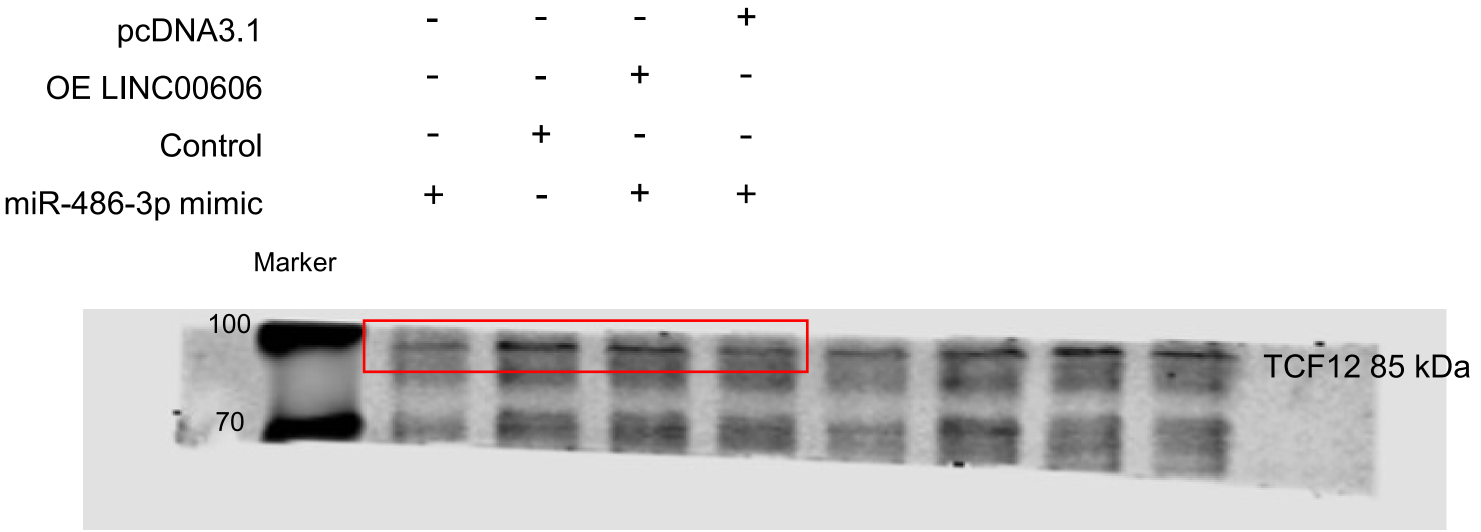

pcDNA3.1 - - - +
OE LINC00606 - - + -
Control - + - -
miR-486-3p mimic + - + +
Marker
100
70
TCF12 85 kDa

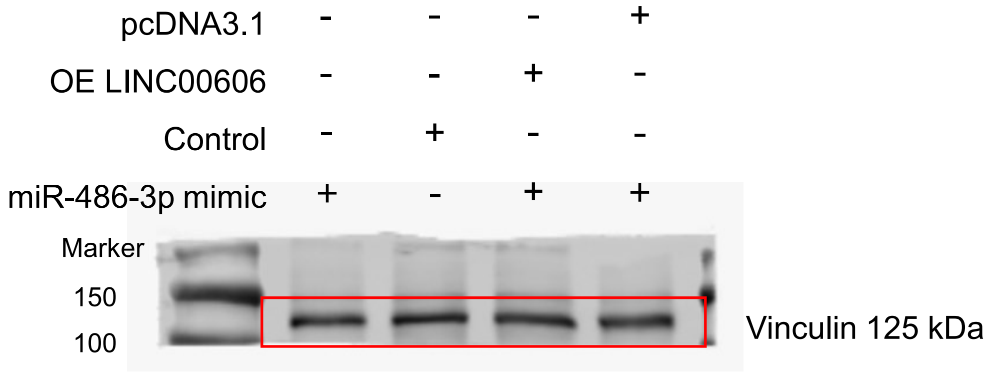

pcDNA3.1 - - - +
OE LINC00606 - - + -
Control - + - -
miR-486-3p mimic + - + +
Marker
150
100
Vinculin 125 kDa

Fig 4 a

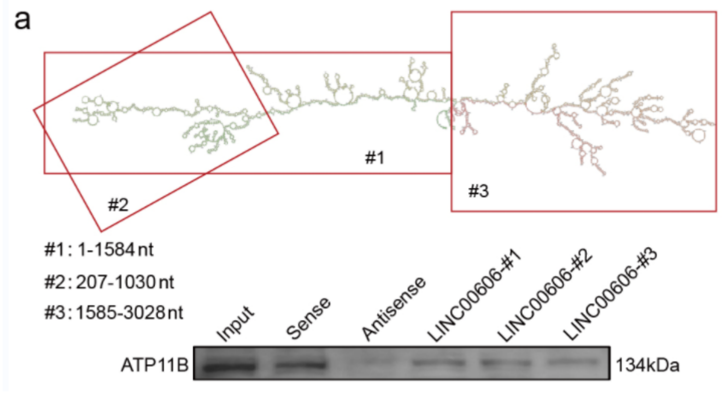
a
#1
#2
#3
#1: 1-1584nt
#2: 207-1030nt
#3: 1585-3028nt
Input
Sense
Antisense
LINC00606-#1
LINC00606-#2
LINC00606-#3
ATP11B
134kDa

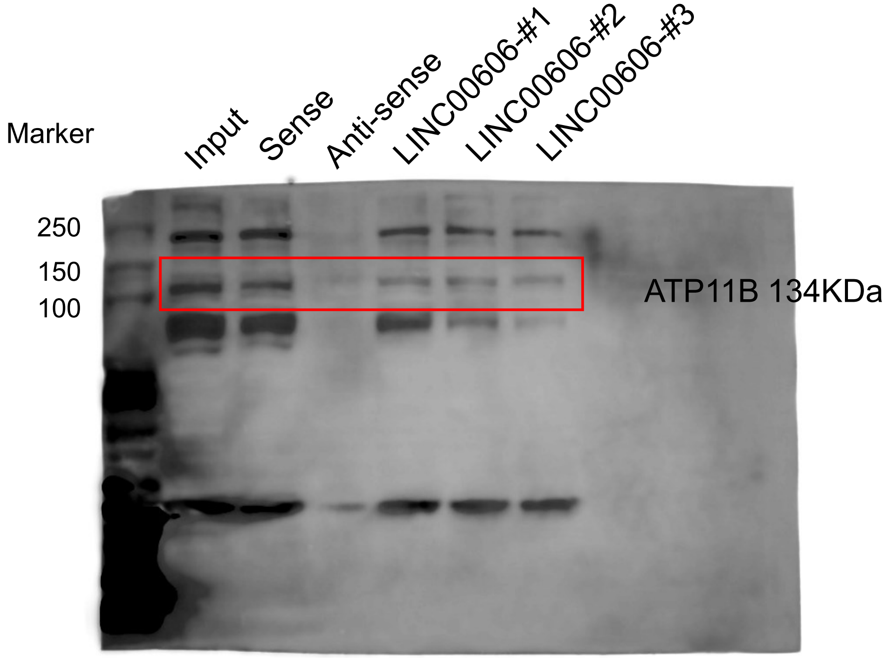
Marker
Input
Sense
Anti-sense
LINC00606-#1
LINC00606-#2
LINC00606-#3
250
150
100
ATP11B 134KDa

Fig 4 d

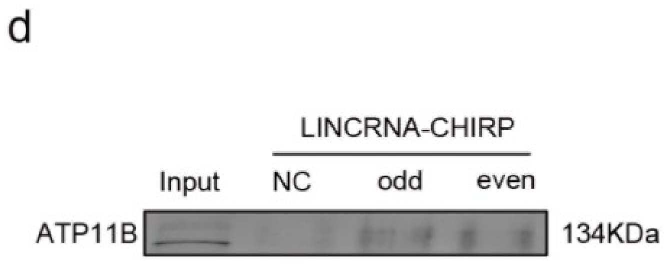
d
LINCRNA-CHIRP
Input
NC
odd
even
ATP11B
134KDa

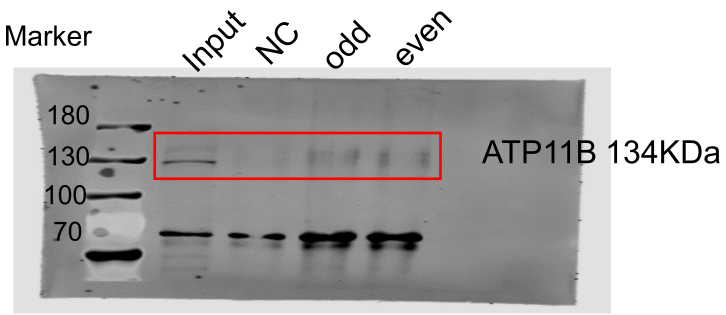
Marker
Input
NC
odd
even
180
130
100
70
ATP11B 134KDa

Fig 4 j

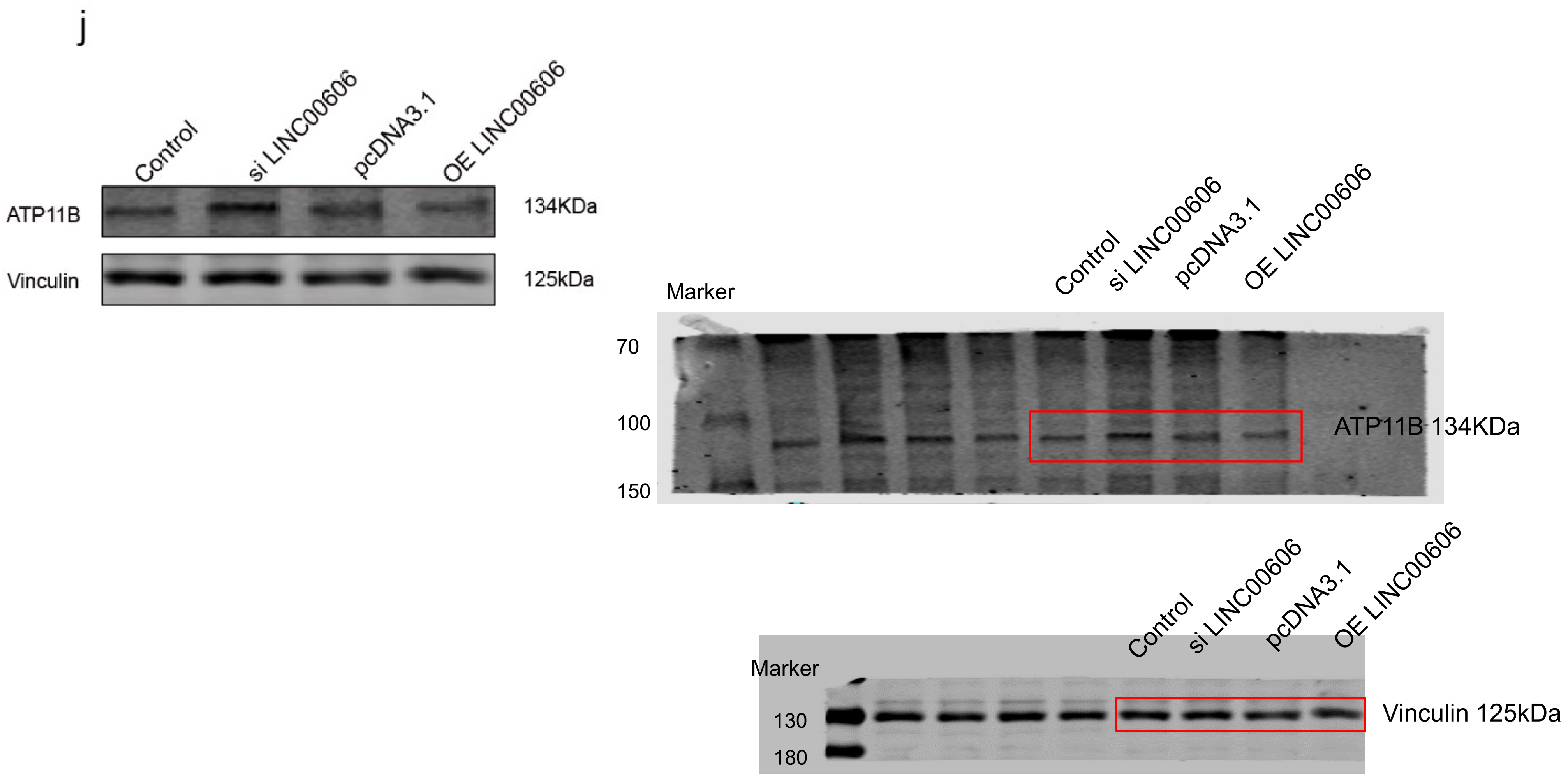

j
Control
si LINC00606
pcDNA3.1
OE LINC00606
ATP11B
134KDa
Vinculin
125kDa
Marker
70
100
150
ATP11B 134KDa
130
180
Vinculin 125kDa

Fig 5 p

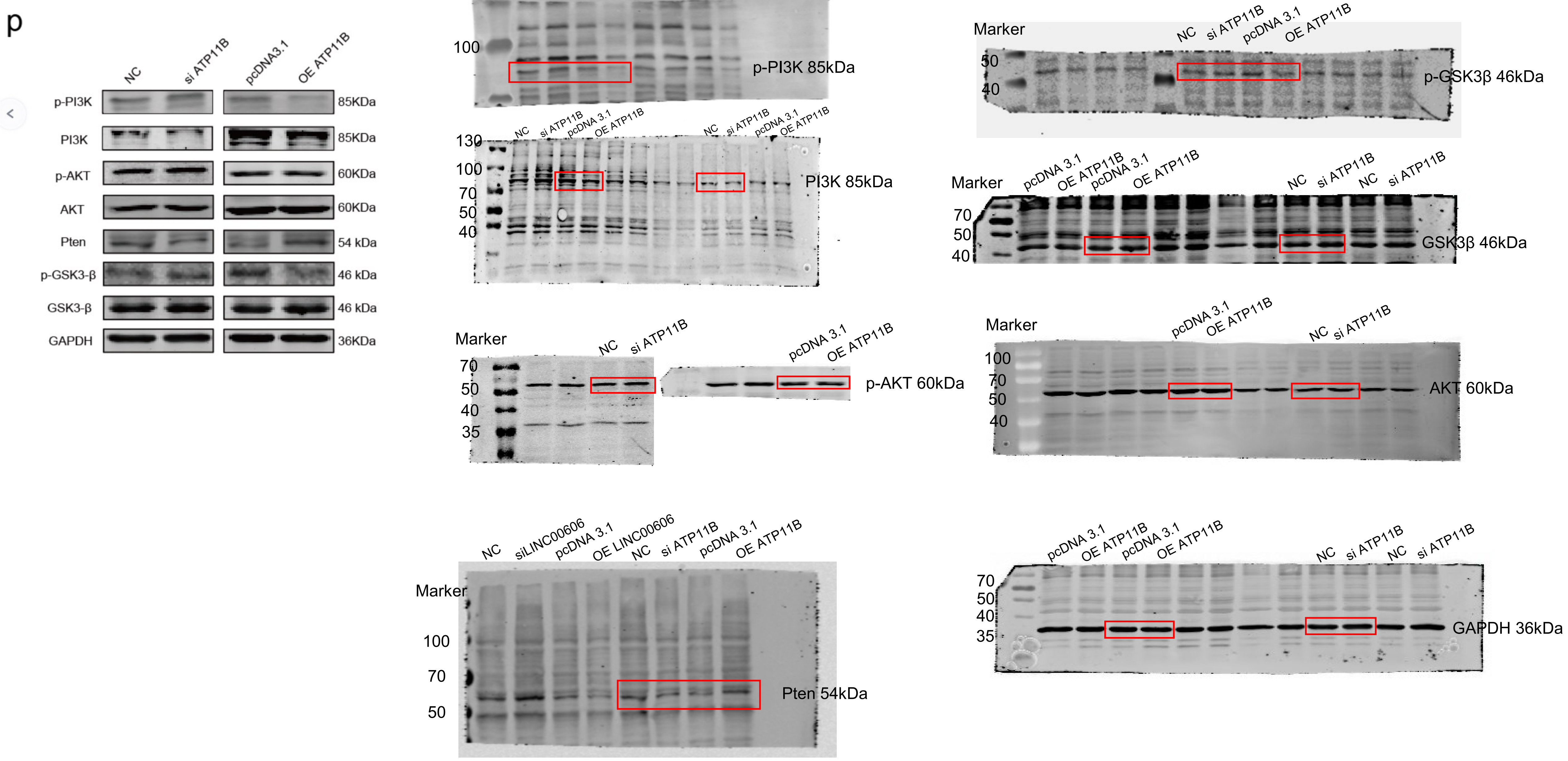

p
NC
si ATP11B
pcDNA3.1
OE ATP11B
p-PI3K
PI3K
p-AKT
AKT
Pten
p-GSK3-β
GSK3-β
GAPDH
85KDa
85KDa
60KDa
60KDa
54 kDa
46 kDa
46 kDa
36KDa
Marker
NC si ATP11B pcDNA 3.1 OE ATP11B
100
p-PI3K 85kDa
NC si ATP11B pcDNA 3.1 OE ATP11B NC si ATP11B pcDNA 3.1 OE ATP11B
130
100
70
50
40
PI3K 85kDa
Marker
NC si ATP11B pcDNA 3.1 OE ATP11B
50
40
p-GSK3β 46kDa
Marker
pcDNA 3.1 OE ATP11B pcDNA 3.1 OE ATP11B NC si ATP11B NC si ATP11B
70
50
40
GSK3β 46kDa
Marker
NC si ATP11B
pcDNA 3.1 OE ATP11B
70
50
40
35
p-AKT 60kDa
Marker
pcDNA 3.1 OE ATP11B NC si ATP11B
100
70
50
40
AKT 60kDa
NC siLINC00606 pcDNA 3.1 OE LINC00606 NC si ATP11B pcDNA 3.1 OE ATP11B
Marker
100
70
50
Pten 54kDa
pcDNA 3.1 OE ATP11B pcDNA 3.1 OE ATP11B NC si ATP11B NC si ATP11B
70
50
40
35
GAPDH 36kDa

Fig 5 q

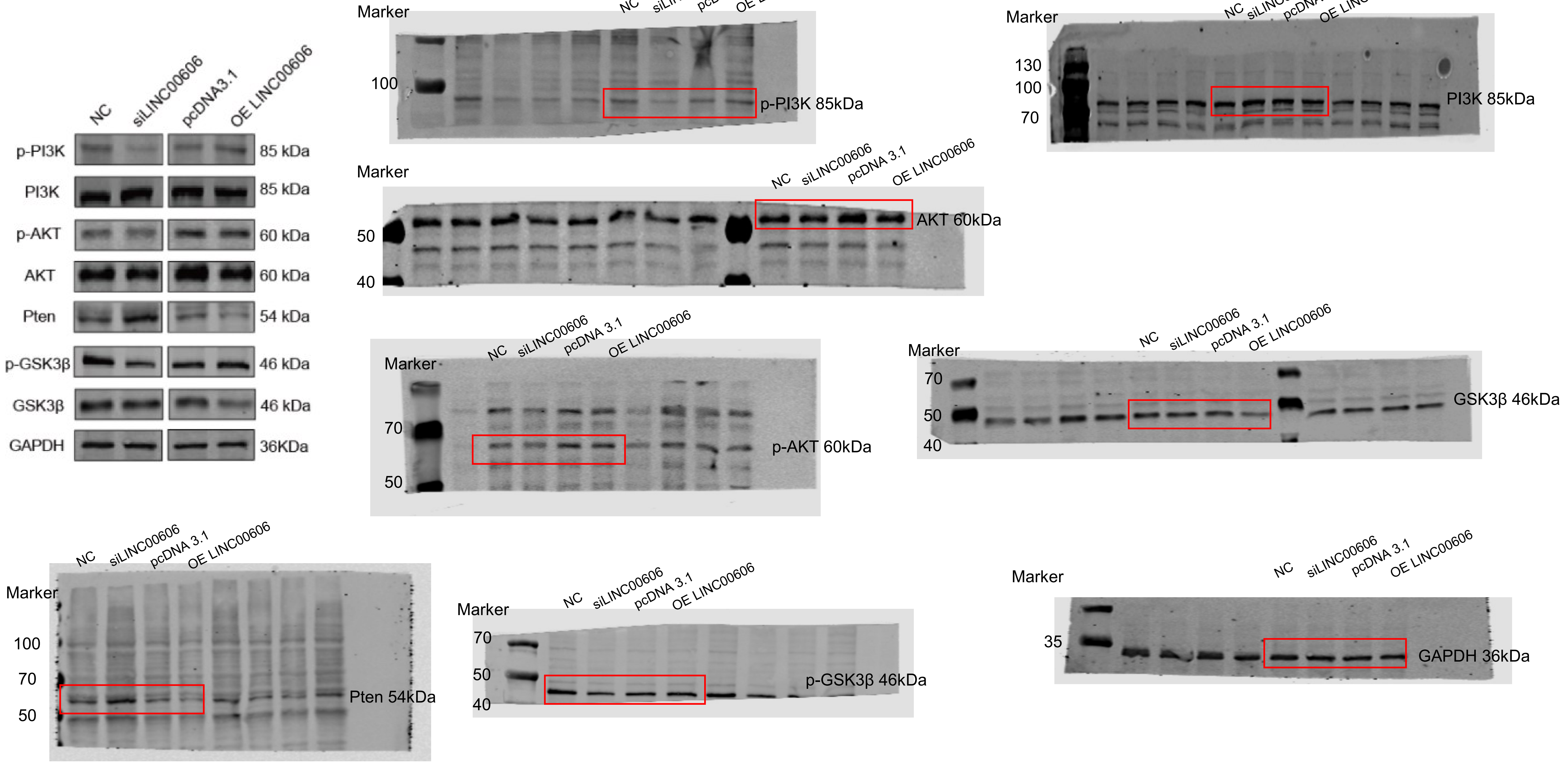

NC
siLINC00606
pcDNA3.1
OE LINC00606
p-PI3K
85 kDa
PI3K
85 kDa
p-AKT
60 kDa
AKT
60 kDa
Pten
54 kDa
p-GSK3β
46 kDa
GSK3β
46 kDa
GAPDH
36KDa
Marker
NC
siLINC00606
pcDNA 3.1
OE LINC00606
100
p-PI3K 85kDa
Marker
130
100
70
PI3K 85kDa
Marker
50
40
AKT 60kDa
Marker
70
50
p-AKT 60kDa
Marker
70
50
40
GSK3β 46kDa
Marker
100
70
50
Pten 54kDa
Marker
70
50
40
p-GSK3β 46kDa
Marker
35
GAPDH 36kDa

Fig 5 r

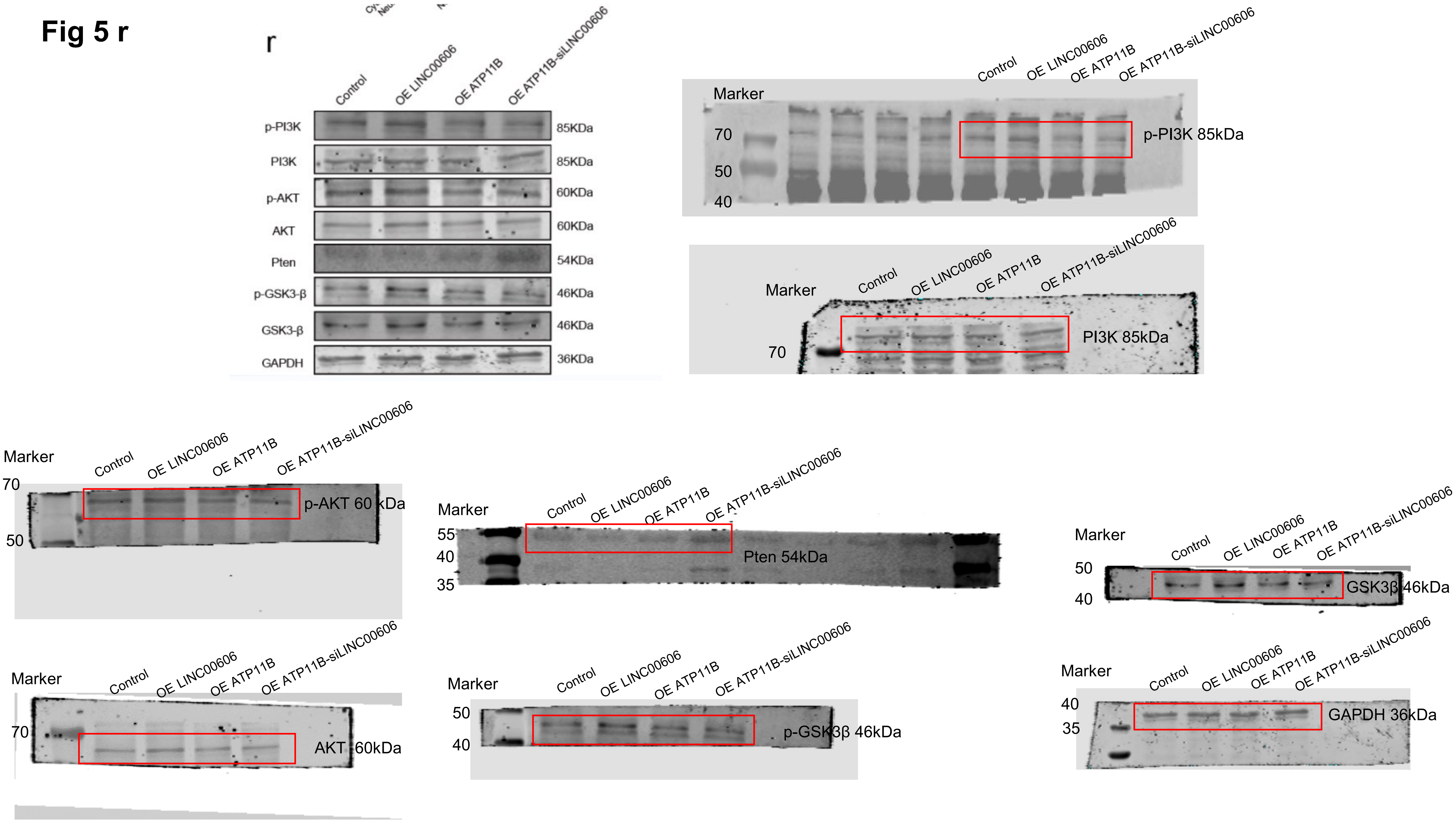

r
Control
OE LINC00606
OE ATP11B
OE ATP11B-siLINC00606
p-PI3K
PI3K
p-AKT
AKT
Pten
p-GSK3-β
GSK3-β
GAPDH
85KDa
85KDa
60KDa
60KDa
54KDa
46KDa
46KDa
36KDa
Marker
70
50
40
p-PI3K 85kDa
Marker
70
PI3K 85kDa
Marker
70
50
p-AKT 60 kDa
Marker
55
40
35
Pten 54kDa
Marker
50
40
GSK3β 46kDa
Marker
70
AKT 60kDa
Marker
50
40
p-GSK3β 46kDa
Marker
40
35
GAPDH 36kDa

Fig 6 i

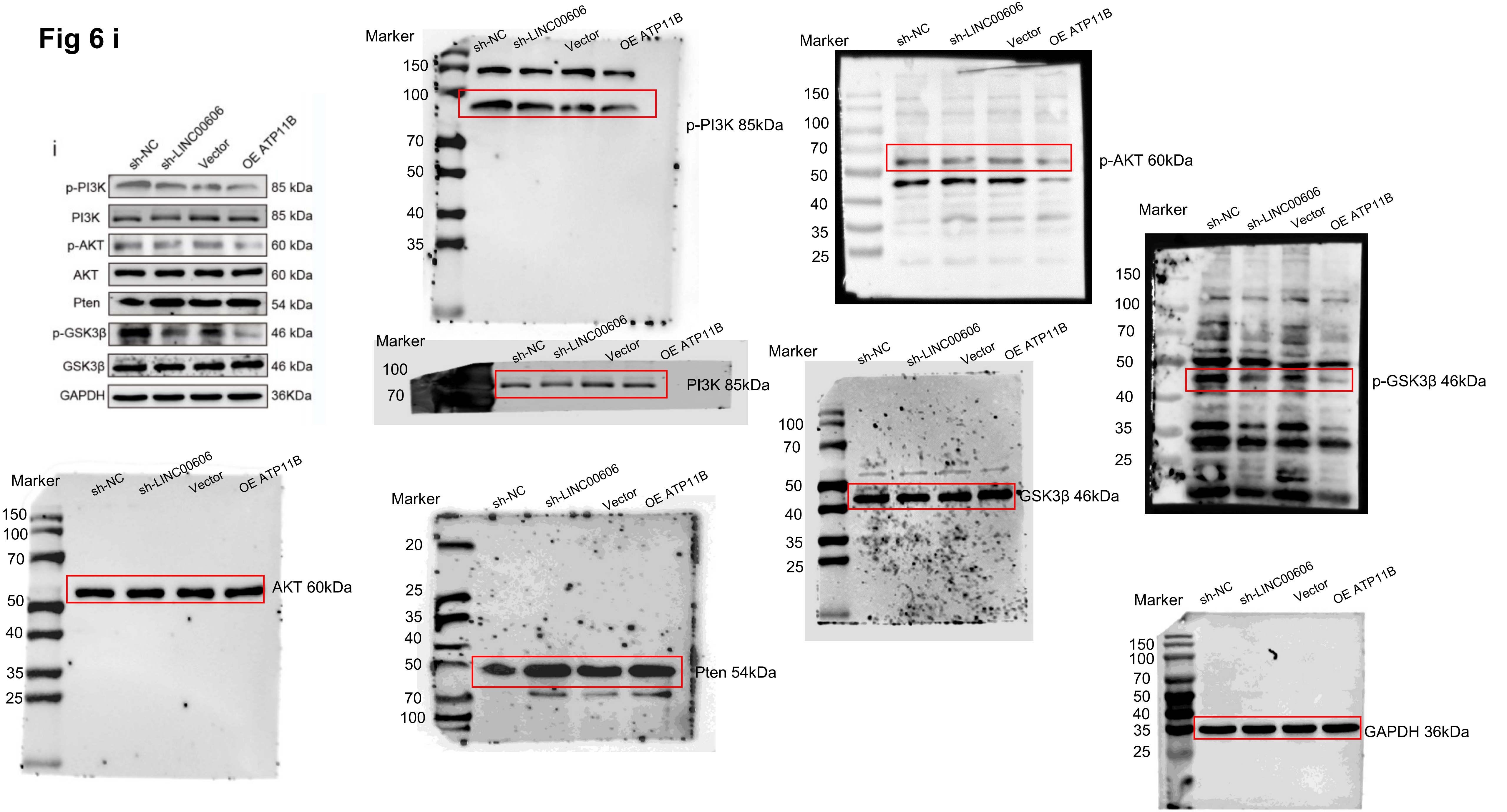

i
sh-NC
sh-LINC00606
Vector
OE ATP11B
p-PI3K
85 kDa
PI3K
85 kDa
p-AKT
60 kDa
AKT
60 kDa
Pten
54 kDa
p-GSK3β
46 kDa
GSK3β
46 kDa
GAPDH
36KDa
Marker
150
100
70
50
40
35
p-PI3K 85kDa
PI3K 85kDa
p-AKT 60kDa
25
p-GSK3β 46kDa
GSK3β 46kDa
AKT 60kDa
20
Pten 54kDa
GAPDH 36kDa

Supplementary Fig S2 b

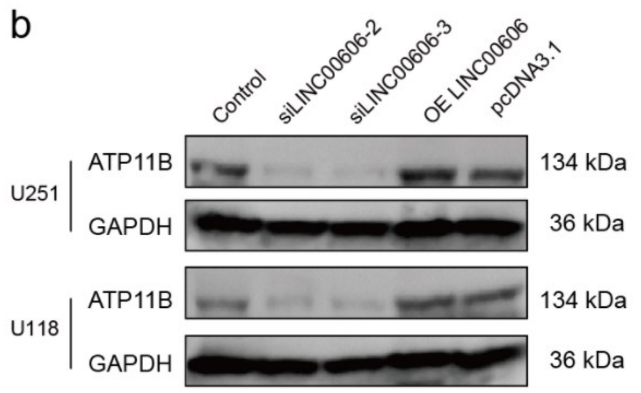

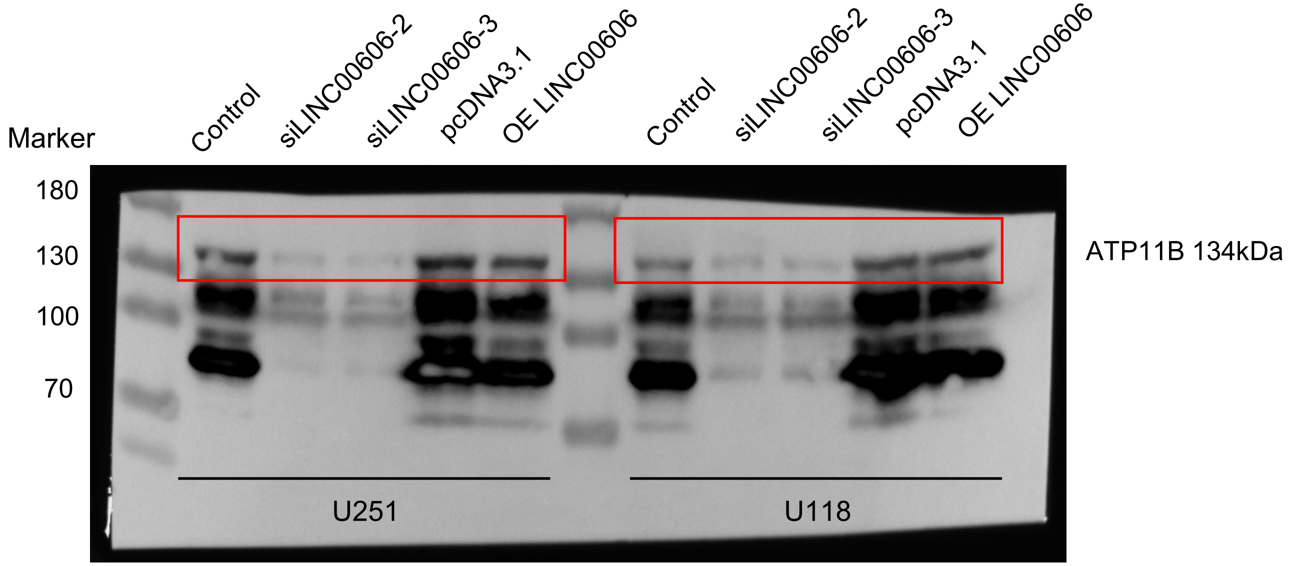

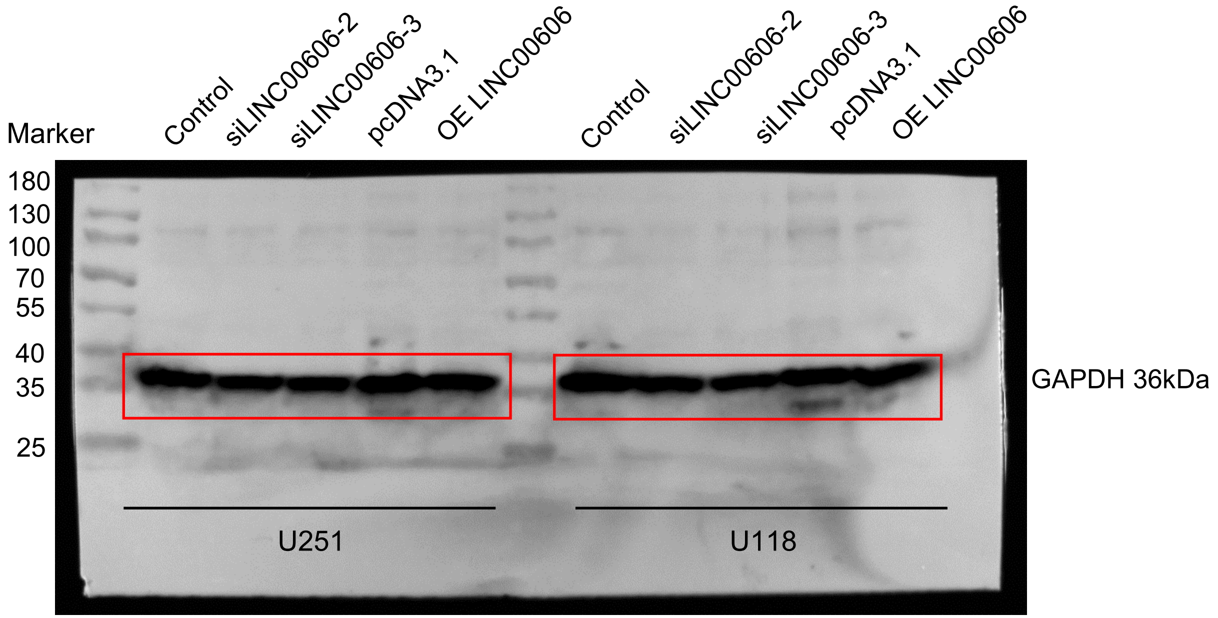

Supplement: Supplementary file 2 — Supplementary Material 2. [file 13046_2024_3058_MOESM2_ESM.pdf]
